# Genetic predisposition to thyrotoxicosis and onset of knee osteoarthritis

**DOI:** 10.3389/fendo.2024.1364027

**Published:** 2024-10-02

**Authors:** Zhiyi He, Zailing Gong, Sizhe Jiao, Wei Xiong, Xiaoxia Hao, Jiarui Cui, Jiaming Zhang

**Affiliations:** ^1^ Department of Orthopedics, Tongji Hospital, Tongji Medical College, Huazhong University of Science and Technology, Wuhan, China; ^2^ Clinical Innovation and Research Center (CIRC), Shenzhen Hospital, Southern Medical University, Shenzhen, China; ^3^ The First Clinical Medical College, Southern Medical University, Guangzhou, China; ^4^ Department of Rehabilitation, Tongji Hospital, Tongji Medical College, Huazhong University of Science and Technology, Wuhan, China

**Keywords:** thyrotoxicosis, hyperthyroidism, Mendelian randomization, risk, osteoarthritis

## Abstract

**Objective:**

Thyroid hormones have actions on cartilage, whereas the association between thyroid hormone related diseases and osteoarthritis (OA) are unclear. This study aims to investigate the association between thyrotoxicosis and OA.

**Methods:**

Summary-level genetic data of thyrotoxicosis were obtained from FinnGen cohorts (nCase = 10,569, nControl = 762,037). Summary-level data of OA were obtained from a large-scale genome-wide association study of UK Biobank (nCase = 40,659, nControl = 756,338). Single nucleotide polymorphisms (SNPs) robustly associated with thyrotoxicosis or OA were used as genetic instruments. A two-sample bidirectional Mendelian randomization (MR) analysis was designed to assess the effect of genetic predisposition of thyrotoxicosis on OA risk, as well as the reverse their relationship. The causal effect was estimated by Inverse-variance weighted method, with weighted median and MR-Egger as supplementary methods.

**Results:**

Genetic predisposition of thyrotoxicosis was associated with the onset of knee OA (autoimmune hyperthyroidism: odds ratio [OR]: 1.05, 95% confidence interval [CI]: 1.03-1.07, FDR < 0.001; thyrotoxicosis: OR: 1.05, 95% CI: 1.02-1.08, FDR = 0.016; thyrotoxicosis with diffuse goitre: OR: 1.04, 95% CI: 1.02-1.07, FDR = 0.003; other and/or unspecified thyrotoxicosis: OR: 1.05, 95% CI: 1.02-1.09, FDR = 0.003), whereas thyrotoxicosis was not associated with hip OA. In reverse MR analysis, genetic predisposition to OA was not associated with thyrotoxicosis. No pleiotropy was identified in the MR analyses. Sensitivity analyses indicated the robustness of the MR estimates.

**Conclusion:**

This study provides MR evidence supporting causal association of thyrotoxicosis with knee OA in European population, whereas OA may have no causal effects on thyrotoxicosis.

## Introduction

Osteoarthritis (OA) is a widespread chronic degenerative joint condition which mainly involves knee joint (1). OA is characterized by the progressive degeneration of articular tissues throughout the entire joint, encompassing not only the cartilage, synovial membrane, and subchondral bone, but also the menisci, infrapatellar fat pad, and the tendons and ligaments (2). In the progression of OA, cartilage degradation (3), synovitis (4), subchondral bone lesions (5), meniscal degeneration (6), inflammation and fibrosis of the infrapatellar fat pad (7), and instability of ligaments (8) not only occur independently but also collaboratively and interactively, either concurrently or sequentially. These pathological changes are interconnected, jointly driving the pathological process of OA, underscoring the systemic nature of this complex disease (2). OA causes chronic pain, swelling, stiffness, and restricted joint movement of patients (9). An epidemiological study from 2020 reported that roughly 3.8% of the global population, equating to 250 million people, are affected by OA (10). Among those over 60 years old, 18% of females and 9.6% of males experience symptomatic OA (10). Beyond the huge health burden on patients, OA also contributes prominently to escalating medical expenses in United States (11). Therefore, early detection and effective intervention are crucial to curtail its prevalence (12, 13). Although some risk factors such as aging (14), trauma (15), obesity (16), and metabolic disorders (17) has been linked to OA, the etiology and pathogenesis have not yet been fully clarified.

The thyroid is a vital endocrine organ responsible for regulating physiological processes through the synthesis and secretion of calcitonin and thyroid hormones (THs), including triiodothyronine (T3) and thyroxine (T4) (18). These hormones are crucial for the normal growth, differentiation, and functioning of various tissues. Insufficient TH levels can result in fatigue, constipation, and weight gain, while excessive levels may heighten the risk of cardiovascular diseases and osteoporosis (19). Thyrotoxicosis refers the clinical manifestation of excessive TH action at the tissue level due to inappropriately elevated concentrations of circulating THs (20). As a subset of thyrotoxicosis, hyperthyroidism specifically denotes the excessive synthesis and secretion of THs by the thyroid gland (20). Graves’ disease, accounting for 70% of cases, is the primary etiology of hyperthyroidism and is defined as autoimmune hyperthyroidism (21). Other causes include toxic nodular hyperthyroidism, constituting 16%, which stems from one or more autonomously functioning thyroid nodules; subacute granulomatous thyroiditis, making up 3%, induced by inflammation; and medications, comprising 9%, notably amiodarone, tyrosine kinase inhibitors, and immune checkpoint inhibitors (22, 23).

While THs are well-established regulators of skeleton development, growth, and maintenance, recent studies also highlight their potential significance in articular cartilage and OA (24). Iodothyronine deiodinase 2 and 3, encoded by DIO2 and DIO3, regulate the conversion between prohormone thyroxine T4 and the bioactive thyroid hormone T3, which were identified as disease susceptibility loci in OA (25, 26). Hyperthyroid states were associated with increased joint effusion and musculoskeletal ultrasound abnormalities (27). Moreover, eprotirome (KB2115), a thyroid hormone receptor (TR) agonist, can cause dose-related articular cartilage damage (28, 29). These studies suggest the potential role of thyroid hormones in OA pathogenesis. However, the association between thyrotoxicosis and the risk of OA is unclear due to no epidemiology evidence.

Mendelian randomization (MR) is an effective method used to elucidate the relationships between exposures and health outcomes (30). By employing genetic variants as proxies for exposures, MR leverages the random allocation of genes at conception. These variants remain unaltered by disease progression, enabling MR to address confounding variables and minimize issues of reverse causation effectively (31, 32). Previous study has suggested the susceptibility loci of thyrotoxicosis (33), which is the basis for using genetic variants as a proxy of thyrotoxicosis. In this study, a bidirectional MR design was utilized to investigate the association between thyrotoxicosis and OA. Genetic variants were used as instrumental proxy of genetic predisposition to thyrotoxicosis, and MR analyses can infer whether OA risk is increased with increased genetic predisposition to thyrotoxicosis. Due to the strength of MR studies, this study offers a causative inference on the causal association between thyrotoxicosis and OA.

## Methods

### Study design

**Figure 1:**
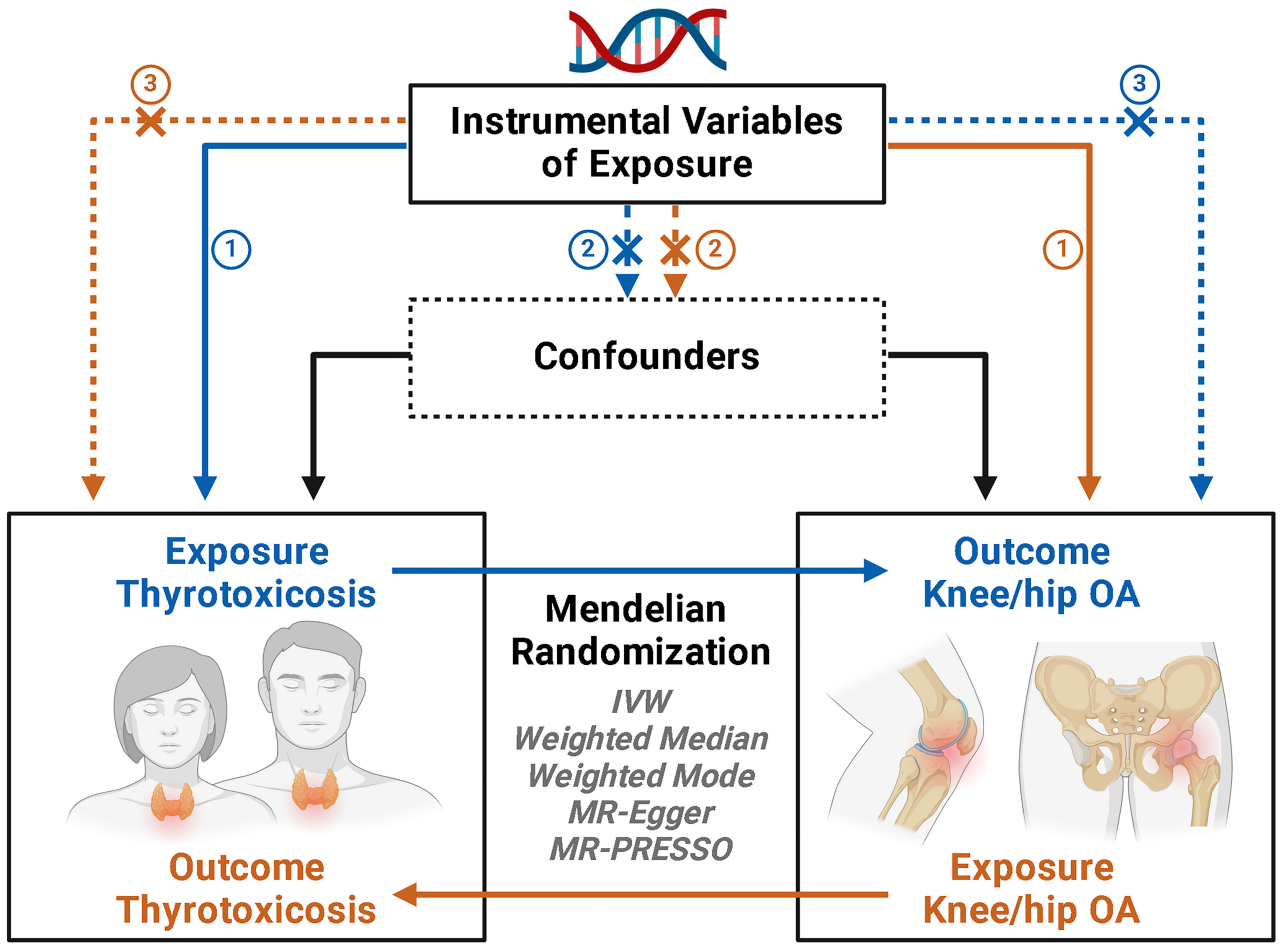
Schematic illustration of the study design.

A two-sample MR analysis to assess the causal impact of thyrotoxicosis on hip or knee OA, utilizing summary-level data of genome-wide association studies (GWAS) from MRC IEU OpenGWAS database (https://gwas.mrcieu.ac.uk/). Three assumptions were satisfied in the MR design: (i) the genetic instruments used as the proxy of genetic predisposition must be robustly correlated with thyrotoxicosis; (ii) the genetic instruments have no association with potential confounders; and (iii) the genetic instruments affect hip or knee OA only through thyrotoxicosis (**Figure 1**). Subsequently, a reverse MR analysis was performed to investigate whether hip or knee OA has a reverse effect on the risk of thyrotoxicosis.

### Data sources for exposure and outcome

Both data sources for exposure and outcome were obtain from MRC IEU OpenGWAS database with GWAS IDs. Summary-level data of thyrotoxicosis-related traits were originally from the FinnGen consortium R5 release, including autoimmune hyperthyroidism (962 cases and 172,976 controls; GWAS ID: “finn-b-AUTOIMMUNE_HYPERTHYROIDISM”), thyrotoxicosis (4,142 cases and 213,693 controls; GWAS ID: “finn-b-THYROTOXICOSIS”), thyrotoxicosis with diffuse goitre (2,350 cases and 187,684 controls; GWAS ID: “finn-b-E4_THYTOXGOITDIF”), and other and/or unspecified thyrotoxicosis (3,115 cases and 187,684 controls; GWAS ID: “finn-b-E4_THYTOXNAS”). Summary-level data of OA-related traits were originally from a genome-wide meta-analysis of UK Biobank (34), including knee OA (2,4955 cases and 378,169 controls; GWAS ID: “ebi-a-GCST007090”) and hip OA (15,704 cases and 378,169 controls; GWAS ID: “ebi-a-GCST007091”). Since the traits of thyrotoxicosis and OA are based on Finland and UK individuals, respectively, there was no overlap between exposure and outcome data sources. All the summary-level data was derived from on both genders of European ancestry. Original studies included in the cited GWASs and consortia received ethical approval from their respective institutional review boards, and all participants had given informed consent. Detailed information on data sources was summarized in **Table 1**.

**Table 1:** Main characteristics of included GWASs.

Traits	Case	Control	Gender	Ancestry	Number of SNPs	GWAS ID
Autoimmune hyperthyroidism	962	172976	Both	European	16380189	finn-b-AUTOIMMUNE_HYPERTHYROIDISM
Thyrotoxicosis	4142	213693	Both	European	16380464	finn-b-THYROTOXICOSIS
Thyrotoxicosis with diffuse goitre	2350	187684	Both	European	16380368	finn-b-E4_THYTOXGOITDIF
Thyrotoxicosis, other and/or unspecified	3115	187684	Both	European	16380371	finn-b-E4_THYTOXNAS
Knee osteoarthritis	24955	378169	Both	European	29999696	ebi-a-GCST007090
Hip osteoarthritis	15704	378169	Both	European	29771219	ebi-a-GCST007091

GWAS, genome-wide association study; SNP, single nucleotide polymorphism.

### Genetic instrument selection

The proxy instruments of genetic predisposition of thyrotoxicosis or OA were selected by rigorous filtering steps to guarantee the assumptions of MR analysis. To select independent and significant genetic instruments, single nucleotide polymorphisms (SNPs) were selected at the genome-wide significance level (P < 5.0×10^-8^) and at linkage disequilibrium (LD) r^2^ < 0.001 within a 10,000-kilobase window based on European ancestry reference data from the 1000 Genomes Project. Then, the selected SNPs were checked by the PhenoScanner platform (https://www.phenoscanner.medschl.cam.ac.uk) to exclude the SNPs associated with potential risk factors of outcome. Third, the SNPs associated with the outcome at the genome-wide significance level (P < 5×10^-5^) were discarded to guarantee the third assumption. Fourth, ambiguous and palindromic SNPs were excluded by harmonizing the SNPs extracted from exposure and outcome data. Fifth, to quantify the strength of the selected genetic instruments, F-statistics was calculated and the SNPs with an F-statistic less than 10 were excluded to avoid weak instrument bias. Finally, Setiger test was performed to remove the SNPs with a reverse causality between the exposure and the outcome. Finally, MR analyses were performed if more than three SNPs were retained.

### Mendelian randomization

The MR analyses were performed with R software (version 4.2.2), TwoSampleMR (version 0.5.6), Mendelian Randomization (version 0.5.0), and MR-PRESSO package. Four MR methods, including inverse variance weighting (IVW), weighted median, weighted mode, and MR-Egger, were used as the previous study descripted (32). IVW method under the random-effect model was used as the primary analysis method, since IVW provides the most precise estimation of causal association. Other MR analyses, such as weighted median and MR-Egger, have been implemented as a complement to IVW. The IVW estimates were compared with weighted median and MR-Egger to check whether the results of different methods were consistent. Potential heterogeneity of SNPs was evaluated using Cochrane’s Q-statistics. MR-Egger intercept was used to identify possible pleiotropic effects, and the MR estimates is invalid if pleiotropy exists. Finally, the robustness of MR estimates was assessed by leave-one-out sensitivity analysis to check whether the MR estimates were driven by any single SNP. After adjusted by Benjamini and Hochberg method for multiple tests, the MR estimates with a FDR < 0.05 was considered statistically significant.

## Results

After the rigorous screening, 5, 9, 10, and 8 SNPs with P < 5.0×10^-8^ and F > 10 were selected to instrument autoimmune hyperthyroidism, thyrotoxicosis, thyrotoxicosis with diffuse goitre, and other and/or unspecified thyrotoxicosis. Moreover, 5 and 23 SNPs with P < 5.0×10^-8^ and F > 10 were selected to instrument knee and hip OA. In the MR analysis of the effect of knee OA on autoimmune hyperthyroidism, one SNP (rs1078301) was excluded by Setiger test. Similarly, two SNPs (rs12040949 and rs4252548) were excluded by Setiger test in the analysis of effect of hip OA on autoimmune hyperthyroidism. The detailed information on genetic instruments selected for thyrotoxicosis and OA-related traits was summarized in **Supplementary Tables 1**–**3**.

**Figure 2:**
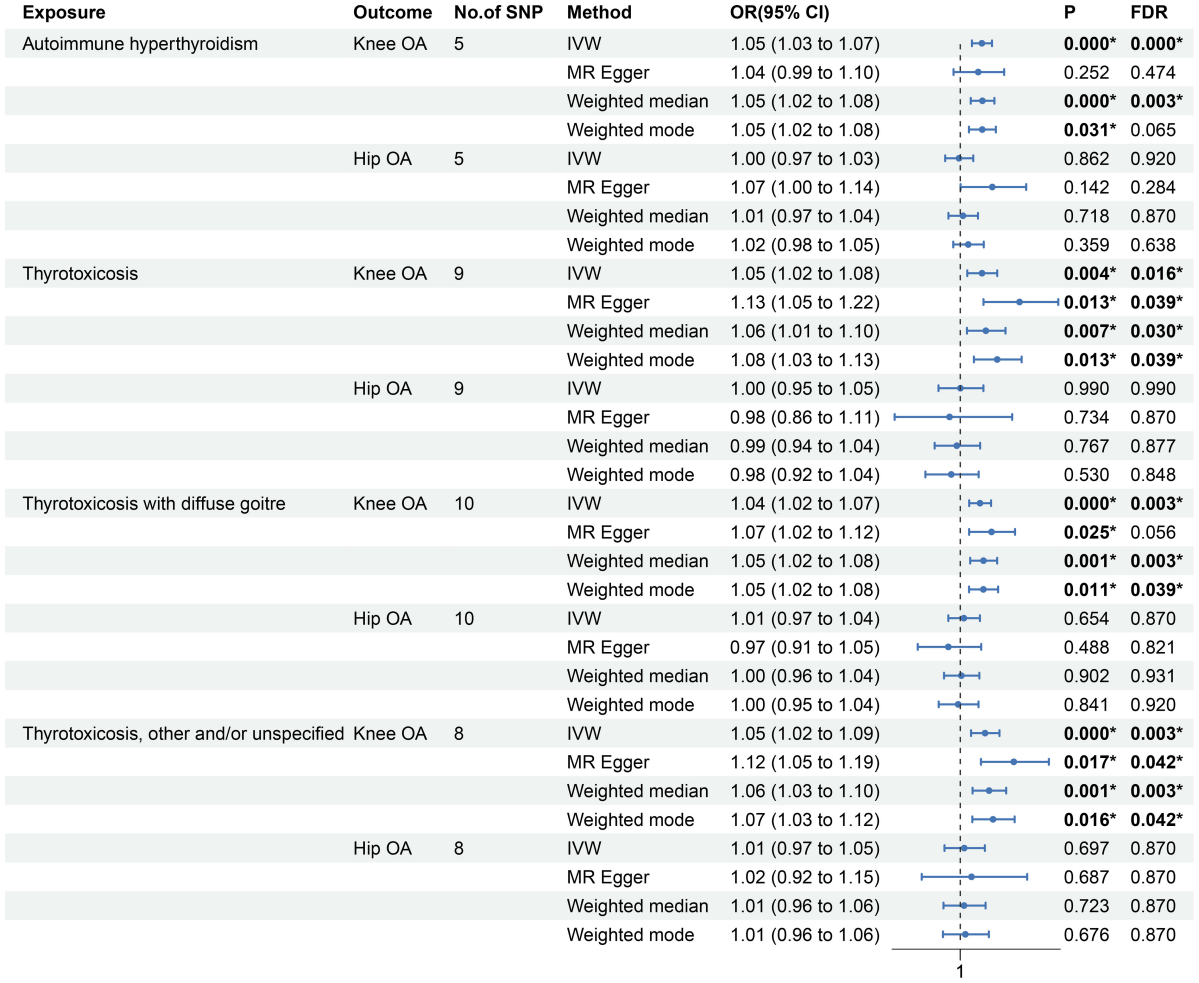
Forest plot of the effect sizes of thyrotoxicosis-OA MR analysis.

As shown in **Figure 2** and **Supplementary Figure 1**, the MR estimates of IVW method indicates that the genetic predisposition of thyrotoxicosis was causally associated with the increased risk of knee OA (autoimmune hyperthyroidism: odds ratio [OR]: 1.05, 95% confidence interval [CI]: 1.03-1.07, P < 0.001, FDR < 0.001; thyrotoxicosis: OR: 1.05, 95% CI: 1.02-1.08, P = 0.004, FDR = 0.016; thyrotoxicosis with diffuse goitre: OR: 1.04, 95% CI: 1.02-1.07, P < 0.001, FDR = 0.003; other and/or unspecified thyrotoxicosis: OR: 1.05, 95% CI: 1.02-1.09, P < 0.001, FDR = 0.003). In the other MR methods, most of MR estimates were statistically significant with a OR more than 1. Since all the P values of Egger intercept were large than 0.05 (**Table 2**), no horizontal pleiotropy was identified in the MR analyses. Thus, the significant MR estimates were valid. Moreover, all P-heterogeneity values of heterogeneity test were not statistically significant, thus no heterogeneity was identified in the IVW and MR Egger analyses (**Table 2**), in line with the funnel plots (**Supplementary Figure 3**). Of note, the P-heterogeneity value of MR Egger is 0.05 in the analysis of thyrotoxicosis and hip OA (**Table 2**). Leave-one-out sensitivity analysis showed that the pooled effect sizes of thyrotoxicosis-OA were all statistically significant after removing a SNP in turn, which indicates the robustness of these associations (**Supplementary Figure 5**). Together, these results suggest a causal association of thyrotoxicosis with knee OA, while no association between thyrotoxicosis and hip OA was identified since all the MR estimates was not significant.

**Table 2:** Thyrotoxicosis-OA MR analysis of for outliers, heterogeneity, and pleiotropy of genetic instruments.

Exposure	Outcome	Method	P-Global test	P-heterogeneity	Egger intercept	P-intercept
Autoimmune hyperthyroidism	Knee OA	IVW		0.854		
		MR Egger		0.742	0.005	0.777
		MR-PRESSO	0.885			
	Hip OA	IVW		0.241		
		MR Egger		0.927	-0.046	0.111
		MR-PRESSO	0.344			
Thyrotoxicosis	Knee OA	IVW		0.314		
		MR Egger		0.736	-0.021	0.061
		MR-PRESSO	0.292			
	Hip OA	IVW		0.073		
		MR Egger		0.050	0.007	0.708
		MR-PRESSO	0.121			
Thyrotoxicosis with diffuse goitre	Knee OA	IVW		0.319		
		MR Egger		0.346	-0.009	0.288
		MR-PRESSO	0.414			
	Hip OA	IVW		0.109		
		MR Egger		0.127	0.012	0.315
		MR-PRESSO	0.138			
Thyrotoxicosis	Knee OA	IVW		0.626		
		MR Egger		0.340	-0.019	0.109
		MR-PRESSO	0.359			
	Hip OA	IVW		0.134		
		MR Egger		0.090	-0.005	0.775
		MR-PRESSO	0.204			

The heterogeneity test in the IVW methods was performed using Cochran’s Q statistic and the global test for the MR-PRESSO method. P < 0.05 was considered significant. OA, osteoarthritis; IVW, inverse variance weighted; P-heterogeneity, P value for heterogeneity test; P-intercept, P value for the intercept of MR-Egger regression.

**Figure 3:**
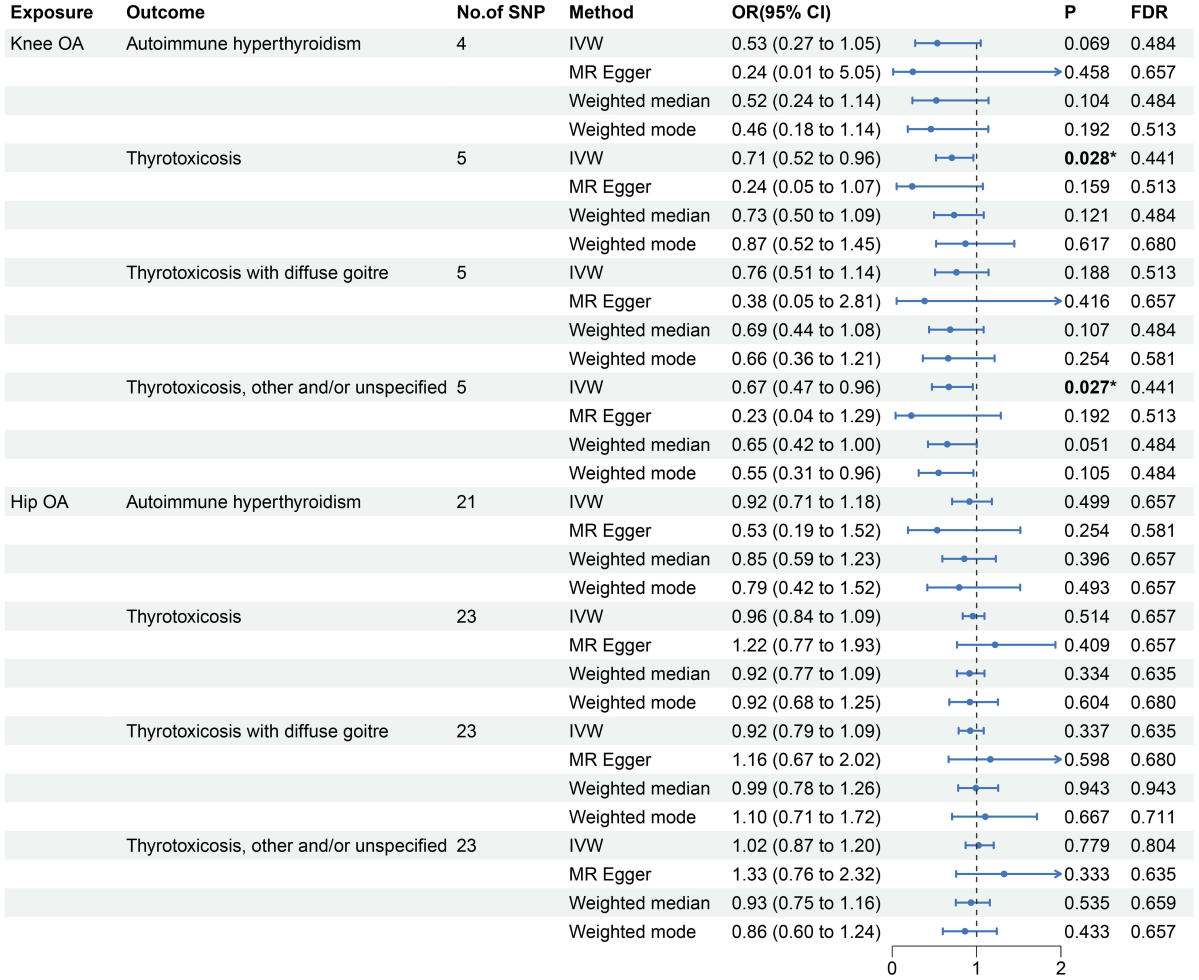
Forest plot of the effect sizes of OA-thyrotoxicosis MR analysis.

The reverse MR analyses indicates that genetic predisposition of OA was not causally associated with thyrotoxicosis (**Figure 3** and **Supplementary Figure 2**). In the MR analysis of knee OA, the results of IVW method showed that knee OA may be associated with thyrotoxicosis (thyrotoxicosis: OR: 0.71, 95% CI: 0.52-0.96, P = 0.028, FDR = 0.441; other and/or unspecified thyrotoxicosis: OR: 0.67, 95% CI: 0.47-0.96, P = 0.027, FDR = 0.441) and the other MR methods also showed a consistent OR with a compromised P value (**Figure 3**). Since all P-intercept are larger than 0.05, no horizontal pleiotropy was identified (**Table 3**). P-Global test and P-heterogeneity of heterogeneity test were not statistically significant, indicating no heterogeneity in the MR analyses (**Table 3**), although some of funnel plots were asymmetry (**Supplementary Figure 4**). Leave-one-out sensitivity analysis indicated the robustness of the associations of knee OA with thyrotoxicosis (**Supplementary Figure 6**). However, since all FDR did not pass the multiple tests, OA appears not associated with thyrotoxicosis.

**Table 3:** OA-thyrotoxicosis MR analysis for outliers, heterogeneity, and pleiotropy of genetic instruments.

Exposure	Outcome	Method	P-Global test	P-heterogeneity	Egger intercept	P-intercept
Knee OA	Autoimmune hyperthyroidism	IVW		0.960		
		MR Egger		0.986	0.056	0.655
		MR-PRESSO	0.944			
	Thyrotoxicosis	IVW		0.679		
		MR Egger		0.976	0.077	0.243
		MR-PRESSO	0.603			
	Thyrotoxicosis with diffuse goitre	IVW		0.808		
		MR Egger		0.769	0.048	0.541
		MR-PRESSO	0.833			
	Thyrotoxicosis	IVW		0.767		
		MR Egger		0.966	0.077	0.299
		MR-PRESSO	0.719			
Hip OA	Autoimmune hyperthyroidism	IVW		0.881		
		MR Egger		0.893	0.051	0.310
		MR-PRESSO	0.871			
	Thyrotoxicosis	IVW		0.222		
		MR Egger		0.233	-0.024	0.293
		MR-PRESSO	0.229			
	Thyrotoxicosis with diffuse goitre	IVW		0.466		
		MR Egger		0.449	-0.022	0.405
		MR-PRESSO	0.444			
	Thyrotoxicosis	IVW		0.131		
		MR Egger		0.133	-0.025	0.353
		MR-PRESSO	0.129			

The heterogeneity test in the IVW methods was performed using Cochran’s Q statistic and the global test for the MR-PRESSO method. P < 0.05 was considered significant. OA, osteoarthritis; IVW, inverse variance weighted; P-heterogeneity, P value for heterogeneity test; P-intercept, P value for the intercept of MR-Egger regression.

## Discussion

The present study employed a two-sample bidirectional MR design to investigate the association between thyrotoxicosis and OA. We found that genetic predisposition of thyrotoxicosis is causally associated with the increased risk of knee OA but not hip OA, while no sufficient evidence support that OA has a reverse effect on thyrotoxicosis onset. These findings provide new insights into thyrotoxicosis and OA.

The role of THs in the development, growth, and maintenance of the skeleton is well-established. The actions of THs are mediated through their interactions with thyroid hormone receptors (TRs), including TRα1, TRβ1 and TRβ2 (35). TRα1 and TRβ1 are present in nearly all tissues, such as cartilage and bone, whereas TRβ2 is primarily expressed in the hypothalamus and pituitary (36, 37). Thyroid hormone transporters (THTs), including monocarboxylate transporters 8 and 10 (MCT8 and MCT10), organic acid transporter protein-1c1 (OATP1c1), and the non-specific L-type amino acid transporters 1 and 2 (LAT1 and LAT2), exhibit varied tissue distribution. Specifically, in the skeleton, MCT10 is predominantly expressed in the growth plate, while MCT8 is found in chondrocytes, osteoblasts, and osteoclasts (24, 38). Additionally, DIO2, essential for converting T4 to T3 in thyroid hormone metabolism, is expressed in chondrocytes and osteoblasts (39, 40). Consequently, the skeleton is a target organ for TH, indicating a potential link between thyroid-related diseases and skeletal disorders, including OA.

The epidemiological evidence linking OA and hyperthyroidism is limited. However, some lines of evidence have indicated their association. DIO2 and DIO3 were identified as OA susceptibility loci (25, 26, 41). DIO2 protein is upregulated in OA cartilage, and the mutation rs225014 of DIO2 gene may be associated with increased OA risk (42). This suggests that DIO2 may be a therapeutic target of OA. Nevertheless, DIO2 also acts as an anti-inflammatory regulator in chondrocytes (40). Inhibiting DIO2 markedly intensifies IL-1β-induced inflammatory responses (40). Therefore, the detailed role of DIO2 remains elucidated and understanding DIO2 levels in the articular cartilage of hyperthyroidism patients is crucial for establishing the connection between DIO2 and OA. Additionally, the TR agonist eprotirome (KB2115) has been shown to induce dose-related articular cartilage damage (28, 29), suggesting that hyperthyroidism-related OA risk might be contingent on the activation of TR downstream signaling.

The potential biological connection between two diseases is subchondral bone lesion. One critical musculoskeletal complication of thyrotoxicosis is osteoporosis. Thyrotoxicosis-related bone loss is primarily attributed to accelerated resorption, which is not offset by a corresponding increase in bone formation. This phenomenon has been predominantly linked to elevated thyroid hormone levels (43). The bone loss associated with high bone turnover may extend to the subchondral bone of joints as well, which is closely linked with the early bone loss and OA onset (5, 44). Elevated TR expression and enhanced nuclear translocation were observed in the osteoblasts of human OA knee (45), indicating that TR signaling is activated in OA osteoblasts. The TR signaling has positive regulation on VEGF expression, a critical factor of angiogenesis inducing OA subchondral bone lesions, since silencing TRα expression decrease VEGF in osteoblasts (45). MMP13, a well-document catabolic factor degrading cartilage, also decreased in chondrocytes when co-cultured with TRα-silenced osteoblasts (45). Thus, treatment with siRNA of TRα succeeded in attenuating subchondral bone loss and cartilage degeneration in OA mouse model (45). Although these lines of evidence suggest a critical role of TR signaling in OA subchondral bone lesions while a more comprehensive study that employs the TH administration into health joint is needed to check whether TH could induce subchondral bone remodeling and OA onset which supports a direction contribution of TH to OA by the actions on joint. Other musculoskeletal symptoms, such as muscular weakness, may contribute to OA. Muscular weakness can increase joint instability, leading to abnormal mechanical loading and subsequent cartilage damage (46).

This study has several limitations. Firstly, the findings of this study are only limited to the European populations due to all the data sources are based on European ancestry. Secondly, since this study is based on summary-level data, the details of the population such as age and sex and the disease-related traits such as duration and treatment are analyzed and the stratification analyses of this factors were not conducted. Thirdly, the associations between THs and OA is not included in this study. It is necessary to treat THs as continuous variables and use nonlinear Mendelian randomization (MR) analysis to explore the relationship between circulating TH concentrations and OA. However, our data source is limited to summary-level data, which restricts our ability to achieve this goal. Finally, while statistically significant in indicating a causal relationship, the association between thyrotoxicosis and OA may be modest. The presence of causal yet small effect sizes underscores the necessity to delve into the underlying mechanisms, offering valuable insights for future research directions. Furthermore, these small effect sizes could bear practical implications, particularly when viewed within a multifactorial framework and considering their cumulative impact. Grasping the role of these factors alongside others is crucial for a comprehensive understanding of OA development or progression.

## Conclusions

Genetic predisposition of thyrotoxicosis is associated with the onset of knee OA but not hip OA in European population, while no sufficient evidence support that OA has a reverse effect on thyrotoxicosis onset. Patients with thyrotoxicosis should be vigilant about joint health and be aware of the potential risk of developing OA.

## Data Availability

The original contributions presented in the study are included in the article/**Supplementary Material**. Further inquiries can be directed to the corresponding authors.

## Supplementary material

Supplementary Table 1 Selection procedures of genetic instruments.

Supplementary Table 2 Genetic instruments proxying genetic predisposition of thyrotoxicosis.

Supplementary Table 3 Genetic instruments proxying genetic predisposition of OA.
