# Supplementary material for: Genetic predisposition to thyrotoxicosis and onset of knee osteoarthritis
Source: Front Endocrinol (Lausanne). 2024 Oct 2;15:1364027. doi: 10.3389/fendo.2024.1364027 (PMC11479908; doi:10.3389/fendo.2024.1364027)
Supplement: Supplementary file 1 [file DataSheet1.pdf]

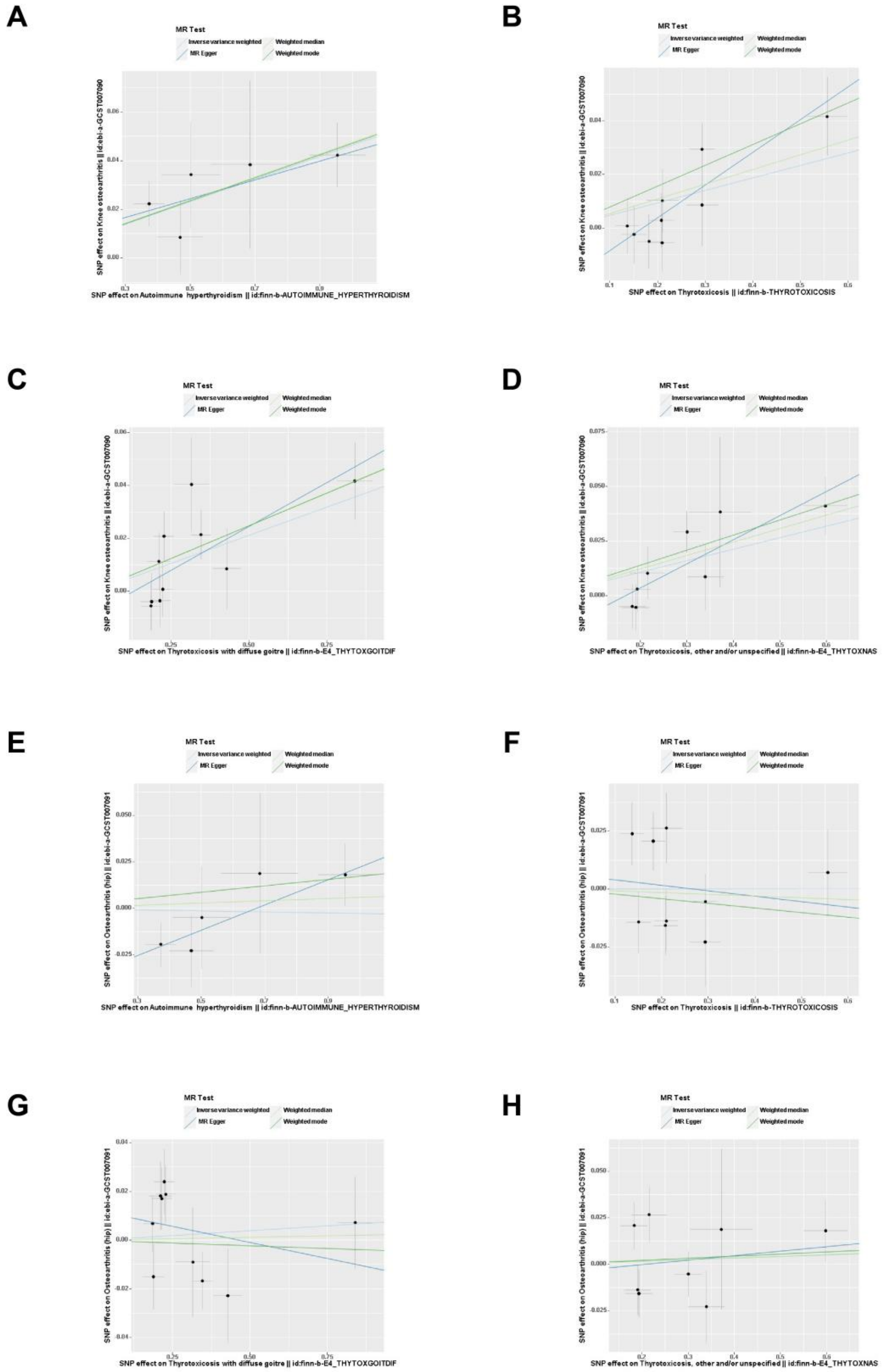

Supplementary Figure 1: Scatter plot of the thyrotoxicosis-OA MR analysis. The scatter plot illustrates the results of a MR analysis investigating the causal effect of autoimmune hyperthyroidism (A, E), thyrotoxicosis (B, F), thyrotoxicosis with diffuse goitre (C, G), thyrotoxicosis (other and/or unspecified) (D, H) on the risk of developing knee or hip OA. The x-axis represents the SNP effect on exposure, and the y-axis represents the SNP effect on outcome. Four different MR methods are shown in the plot, including inverse variance weighted, MR Egger, weighted median, and weighted mode. Each black dot represents an individual SNP, with error bars indicating the standard error of the SNP effect estimates on both traits. The different MR methods' regression lines suggest the overall direction and magnitude of the causal relationship between exposure and outcome.

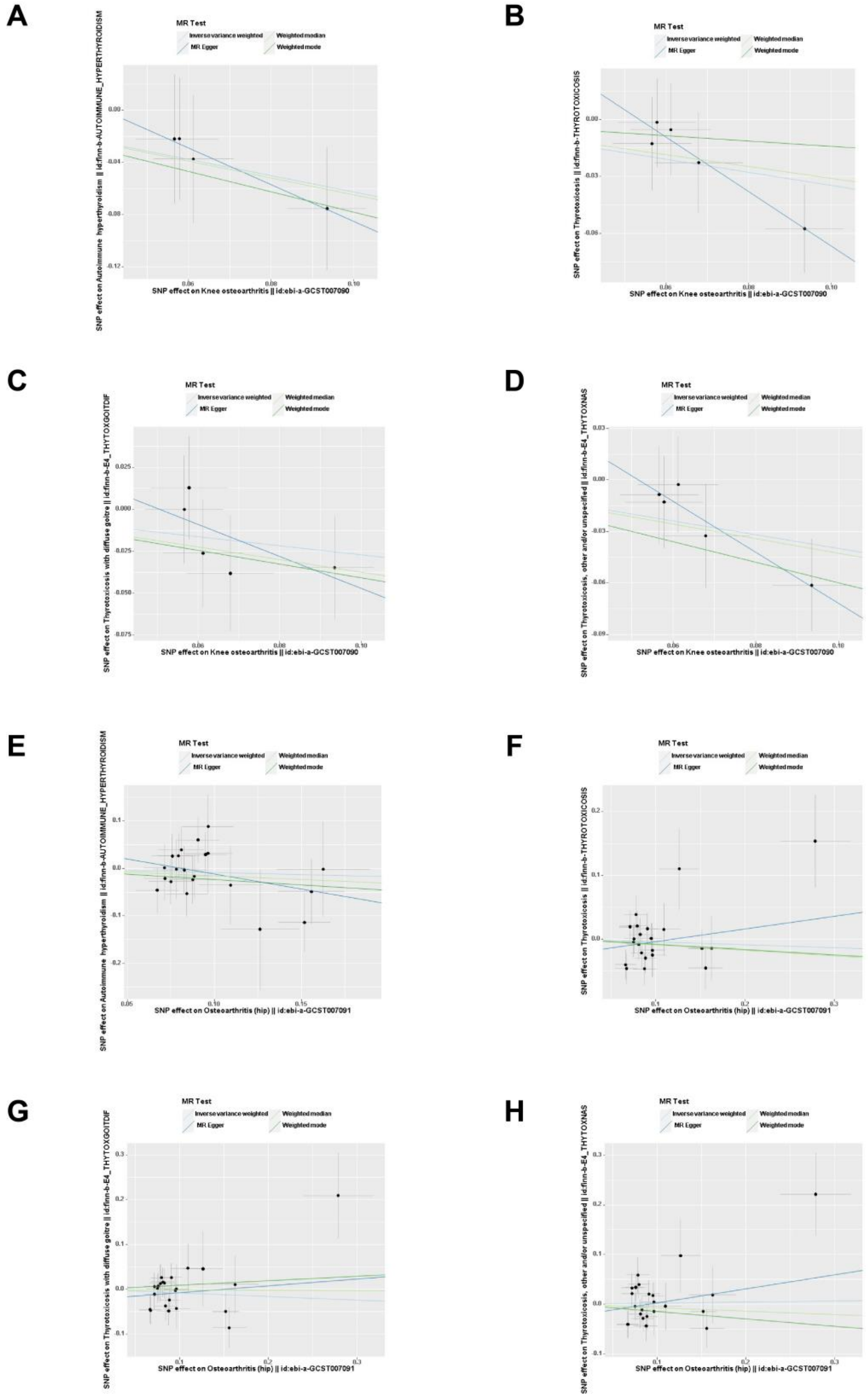

Supplementary Figure 2: Scatter plot of the OA-thyrotoxicosis MR analysis. The scatter plot illustrates the results of a MR analysis investigating the causal effect of knee or hip OA on the risk of developing autoimmune hyperthyroidism (A, E), thyrotoxicosis (B, F), thyrotoxicosis with diffuse goitre (C, G), thyrotoxicosis (other and/or unspecified) (D, H). The x-axis represents the SNP effect on exposure, and the y-axis represents the SNP effect on outcome. Four different MR methods are shown in the plot, including inverse variance weighted, MR Egger, weighted median, and weighted mode. Each black dot represents an individual SNP, with error bars indicating the standard error of the SNP effect estimates on both traits. The different MR methods' regression lines suggest the overall direction and magnitude of the causal relationship between exposure and outcome.

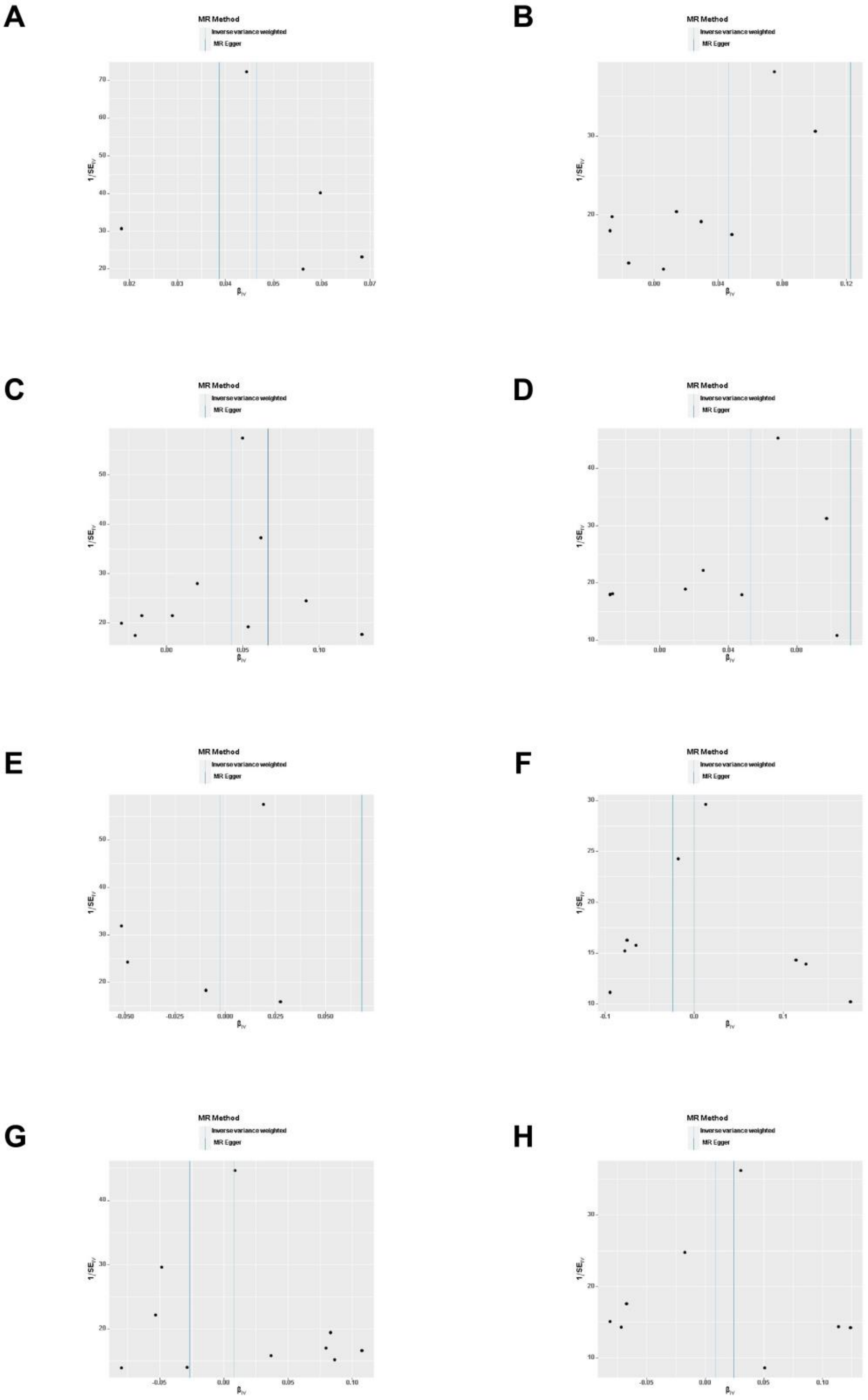

Supplementary Figure 3: Funnel plot of the thyrotoxicosis-OA MR analysis. (A) autoimmune hyperthyroidism-knee OA MR; (B) thyrotoxicosis-knee OA MR; (C) thyrotoxicosis with diffuse goitre-knee OA MR; (D) thyrotoxicosis (other and/or unspecified)-knee OA MR; (E) autoimmune hyperthyroidism-hip OA MR; (F) thyrotoxicosis-hip OA MR; (G) thyrotoxicosis with diffuse goitre-hip OA MR; (H) thyrotoxicosis (other and/or unspecified)-hip OA MR.

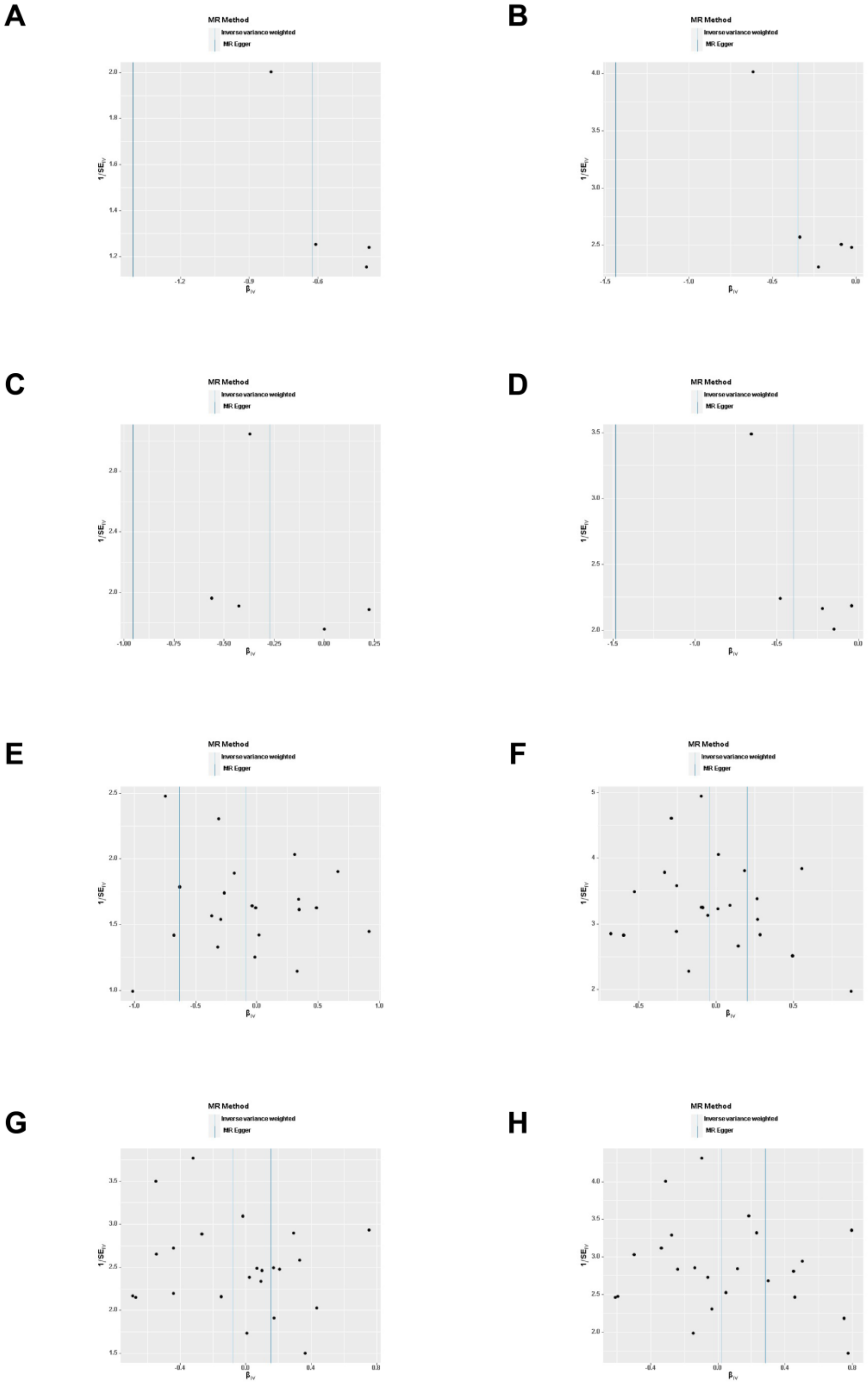

Supplementary Figure 4: Funnel plot of the OA-thyrotoxicosis MR analysis. (A) knee OA-autoimmune hyperthyroidism MR; (B) knee OA-thyrotoxicosis MR; (C) knee OA-thyrotoxicosis with diffuse goitre MR; (D) knee OA-thyrotoxicosis (other and/or unspecified) MR; (E) hip OA-autoimmune hyperthyroidism MR; (F) hip OA-thyrotoxicosis MR; (G) hip OA-thyrotoxicosis with diffuse goitre MR; (H) hip OA-thyrotoxicosis (other and/or unspecified) MR.

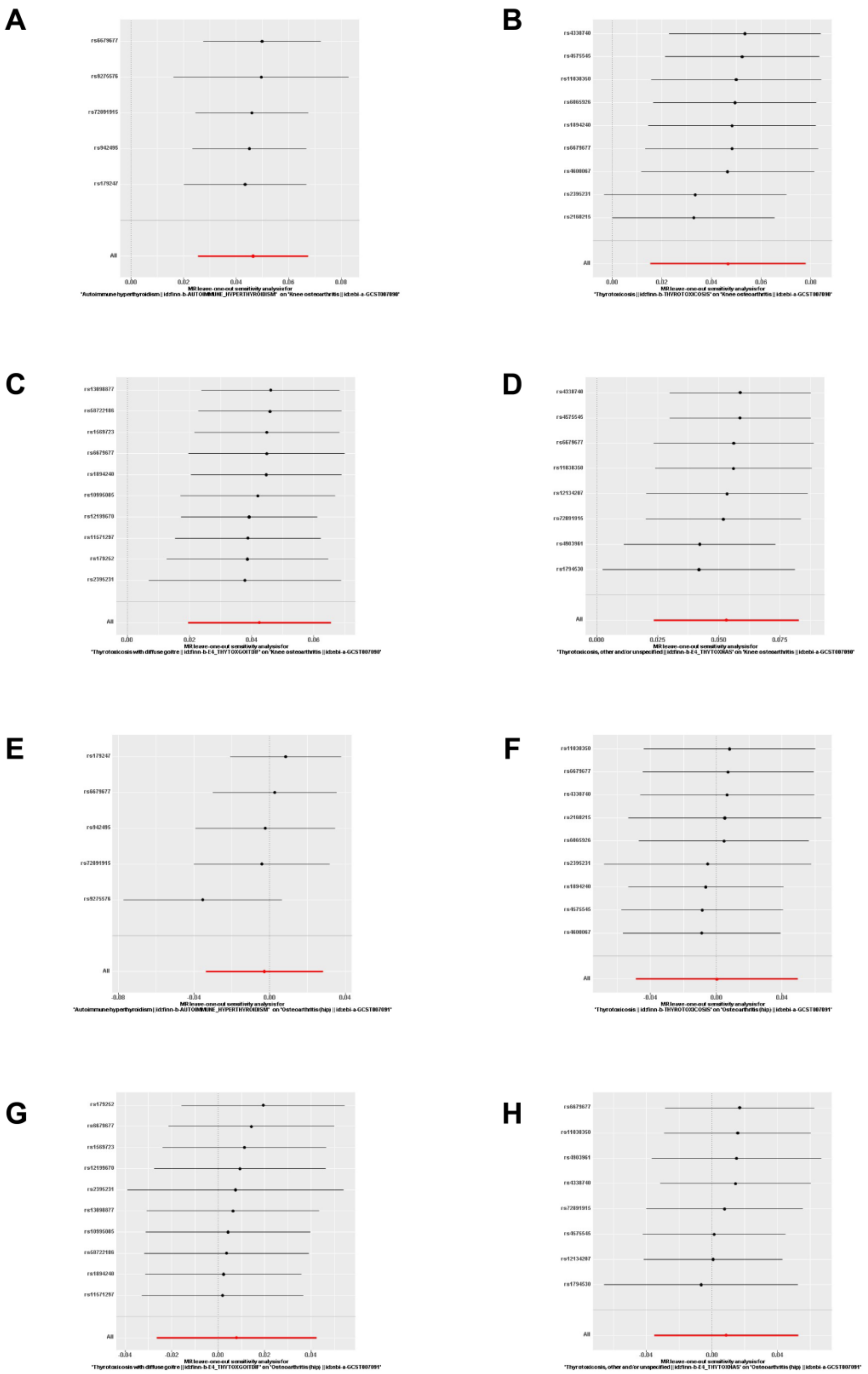

Supplementary Figure 5. Leave-one-out analysis of the thyrotoxicosis-OA MR analysis. (A) autoimmune hyperthyroidism-knee OA MR; (B) thyrotoxicosis-knee OA MR; (C) thyrotoxicosis with diffuse goitre-knee OA MR; (D) thyrotoxicosis (other and/or unspecified)-knee OA MR; (E) autoimmune hyperthyroidism-hip OA MR; (F) thyrotoxicosis-hip OA MR; (G) thyrotoxicosis with diffuse goitre-hip OA MR; (H) thyrotoxicosis (other and/or unspecified)-hip OA MR.

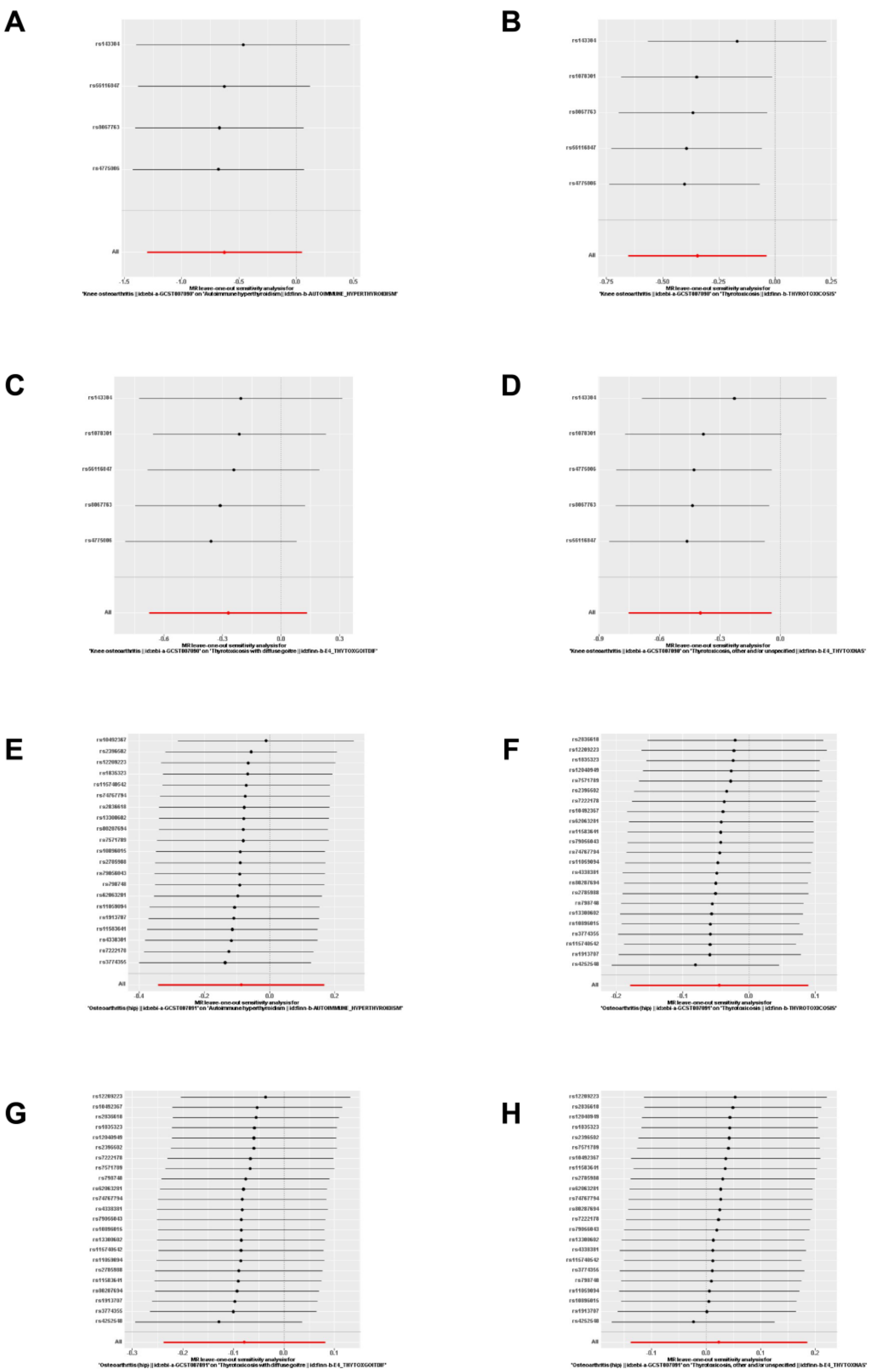

Supplementary Figure 6. Leave-one-out analysis of the OA-thyrotoxicosis MR analysis. (A) knee OA-autoimmune hyperthyroidism MR; (B) knee OA-thyrotoxicosis MR; (C) knee OA-thyrotoxicosis with diffuse goitre MR; (D) knee OA-thyrotoxicosis (other and/or unspecified) MR; (E) hip OA-autoimmune hyperthyroidism MR; (F) hip OA-thyrotoxicosis MR; (G) hip OA-thyrotoxicosis with diffuse goitre MR; (H) hip OA-thyrotoxicosis (other and/or unspecified) MR.

Mendelian randomization	Exposure ID	Outcome ID	SNP number					
			Step 1	Step 2	Step 3	Step 4	Step 5	Step 6
Autoimmune hyperthyroidism->Knee OA	finn-b-AUTOIMMUNE_HYPERTHYROIDISM	ebi-a-GCST007090	7	5	5	5	5	5
Thyrotoxicosis->Knee OA	finn-b-THYROTOXICOSIS	ebi-a-GCST007090	11	10	10	9	9	9
Thyrotoxicosis with diffuse goitre->Knee OA	finn-b-E4_THYTOXGOITDIF	ebi-a-GCST007090	12	11	11	10	10	10
Thyrotoxicosis, other and/or unspecified->Knee OA	finn-b-E4_THYTOXNAS	ebi-a-GCST007090	11	9	9	8	8	8
Autoimmune hyperthyroidism->Hip OA	finn-b-AUTOIMMUNE_HYPERTHYROIDISM	ebi-a-GCST007091	7	5	5	5	5	5
Thyrotoxicosis->Hip OA	finn-b-THYROTOXICOSIS	ebi-a-GCST007091	11	10	10	9	9	9
Thyrotoxicosis with diffuse goitre->Hip OA	finn-b-E4_THYTOXGOITDIF	ebi-a-GCST007091	12	11	11	10	10	10
Thyrotoxicosis, other and/or unspecified->Hip OA	finn-b-E4_THYTOXNAS	ebi-a-GCST007091	11	9	9	8	8	8
Knee OA->Autoimmune hyperthyroidism	ebi-a-GCST007090	finn-b-AUTOIMMUNE_HYPERTHYROIDISM	10	8	7	5	5	4
Knee OA->Thyrotoxicosis	ebi-a-GCST007090	finn-b-THYROTOXICOSIS	10	8	7	5	5	5
Knee OA->Thyrotoxicosis with diffuse goitre	ebi-a-GCST007090	finn-b-E4_THYTOXGOITDIF	10	8	7	5	5	5
Knee OA->Thyrotoxicosis, other and/or unspecified	ebi-a-GCST007090	finn-b-E4_THYTOXNAS	10	8	7	5	5	5
Hip OA->Autoimmune hyperthyroidism	ebi-a-GCST007091	finn-b-AUTOIMMUNE_HYPERTHYROIDISM	27	26	26	23	23	21
Hip OA->Thyrotoxicosis	ebi-a-GCST007091	finn-b-THYROTOXICOSIS	27	26	26	23	23	23
Hip OA->Thyrotoxicosis with diffuse goitre	ebi-a-GCST007091	finn-b-E4_THYTOXGOITDIF	27	26	26	23	23	23
Hip OA->Thyrotoxicosis, other and/or unspecified	ebi-a-GCST007091	finn-b-E4_THYTOXNAS	27	26	26	23	23	23

**Step 1**	Slection: pval.exposure<5e-08, clump_r2=0.001, clump_kb=10000
**Step 2**	SNP extraction from outcome dataset and Check with PhenoScanner
**Step 3**	Exclusion: pval.outcome<5e-05
**Step 4**	Harmonization
**Step 5**	Exclusion: F-statistics<10
**Step 6**	Setiger test

SNP	effect_allele	other_allele	Exposure								Outcome: knee OA						Outcome: Hip OA					
			beta	se	pva	eaf	F-statistics	samplesize	name	id	beta	se	pva	eaf	samplesize	id	beta	se	pva	eaf	samplesize	id
rs179247	G	A	-0.3733	0.0472	2.47E-15	0.5785	1.33E+03	18258	Autoimmune hyperthyroidism	finn-b-AUTOIMMUNE	-0.0223	0.0093	0.01662	0.4799	403124	ebi-a-GCS	0.0193	0.0117	0.09986	0.4804	393873	ebi-a-GCST007091
rs6679677	A	C	0.4689	0.0692	1.20E-11	0.1402	1.02E+03	18258	Autoimmune hyperthyroidism	finn-b-AUTOIMMUNE	0.0086	0.0153	0.575999	0.1016	403124	ebi-a-GCS	-0.0228	0.0193	0.2375	0.1012	393873	ebi-a-GCST007091
rs7289191	A	G	0.6845	0.1204	1.31E-08	0.03931	6.70E+02	18258	Autoimmune hyperthyroidism	finn-b-AUTOIMMUNE	0.0384	0.0344	0.2639	0.0193	403124	ebi-a-GCS	0.0188	0.0431	0.6623	0.0193	393873	ebi-a-GCST007091
rs927557	T	C	0.9534	0.0861	1.70E-28	0.09458	3.37E+03	18258	Autoimmune hyperthyroidism	finn-b-AUTOIMMUNE	0.0423	0.0132	0.001347	0.1451	403124	ebi-a-GCS	0.0181	0.0166	0.2759	0.1447	393873	ebi-a-GCST007091
rs942495	T	C	0.502	0.0903	2.75E-08	0.07115	6.29E+02	18258	Autoimmune hyperthyroidism	finn-b-AUTOIMMUNE	0.0343	0.0217	0.1134	0.05	403124	ebi-a-GCS	-0.0049	0.0274	0.8571	0.0498	393873	ebi-a-GCST007091
rs1103835	G	C	0.2084	0.0253	1.78E-16	0.3069	4.10E+03	217835	Thyrotoxicosis	finn-b-THYROTOXIC	0.0029	0.0102	0.7739	0.2979	403124	ebi-a-GCS	-0.0157	0.0128	0.2194	0.2976	393873	ebi-a-GCST007091
rs189424	G	A	-0.1368	0.0244	1.96E-08	0.5128	2.06E+03	217835	Thyrotoxicosis	finn-b-THYROTOXIC	-8.00E-04	0.0104	0.9376	0.4481	403124	ebi-a-GCS	-0.024	0.0134	0.07443	0.4479	393873	ebi-a-GCST007091
rs2160215	C	T	0.2936	0.0256	1.69E-30	0.2966	8.13E+03	217835	Thyrotoxicosis	finn-b-THYROTOXIC	0.0295	0.0096	0.002098	0.3756	403124	ebi-a-GCS	-0.0053	0.0121	0.6582	0.375	393873	ebi-a-GCST007091
rs2395231	A	G	0.5562	0.0421	9.18E-40	0.09569	1.23E+04	217835	Thyrotoxicosis	finn-b-THYROTOXIC	0.0417	0.0146	0.004308	0.1284	403124	ebi-a-GCS	0.0072	0.0188	0.7021	0.1282	393873	ebi-a-GCST007091
rs433874	C	T	0.2097	0.025	4.77E-17	0.3142	4.21E+03	217835	Thyrotoxicosis	finn-b-THYROTOXIC	-0.0055	0.0106	0.6058	0.2609	403124	ebi-a-GCS	-0.0137	0.0133	0.3017	0.2608	393873	ebi-a-GCST007091
rs4575545	A	G	0.182	0.0252	5.04E-13	0.3039	3.10E+03	217835	Thyrotoxicosis	finn-b-THYROTOXIC	-0.005	0.0101	0.6216	0.3065	403124	ebi-a-GCS	0.0208	0.0127	0.1006	0.3067	393873	ebi-a-GCST007091
rs4600067	G	A	0.2103	0.0333	2.66E-10	0.1411	2.36E+03	217835	Thyrotoxicosis	finn-b-THYROTOXIC	0.0102	0.012	0.3946	0.1847	403124	ebi-a-GCS	0.0264	0.0151	0.07927	0.1843	393873	ebi-a-GCST007091
rs606592	G	A	0.1504	0.0261	8.00E-09	0.7271	1.97E+03	217835	Thyrotoxicosis	finn-b-THYROTOXIC	-0.0024	0.0108	0.8266	0.7553	403124	ebi-a-GCS	-0.0142	0.0135	0.2944	0.7543	393873	ebi-a-GCST007091
rs6679677	A	C	0.2934	0.0331	7.94E-19	0.1471	4.81E+03	217835	Thyrotoxicosis	finn-b-THYROTOXIC	0.0086	0.0153	0.575999	0.1016	403124	ebi-a-GCS	-0.0228	0.0193	0.2375	0.1012	393873	ebi-a-GCST007091
rs1099508	T	C	0.2112	0.0367	8.72E-09	0.2271	3.02E+03	189998	Thyrotoxicosis with diffuse goitre	finn-b-E4_THYTOXG	0.0113	0.011	0.3076	0.2344	403124	ebi-a-GCS	0.0182	0.0139	0.1882	0.2342	393873	ebi-a-GCST007091
rs1157129	C	T	-0.2276	0.0313	3.57E-13	0.3737	4.72E+03	189998	Thyrotoxicosis with diffuse goitre	finn-b-E4_THYTOXG	-0.0208	0.0093	0.0256	0.4889	403124	ebi-a-GCS	-0.0189	0.0117	0.1065	0.4892	393873	ebi-a-GCST007091
rs1219967	C	G	0.3156	0.0558	1.54E-08	0.07769	2.75E+03	189998	Thyrotoxicosis with diffuse goitre	finn-b-E4_THYTOXG	0.0404	0.0179	0.02358	0.0727	403124	ebi-a-GCS	-0.009	0.0225	0.6902	0.0724	393873	ebi-a-GCST007091
rs1309887	T	C	0.1856	0.0304	1.03E-09	0.5206	3.32E+03	189998	Thyrotoxicosis with diffuse goitre	finn-b-E4_THYTOXG	-0.0055	0.0093	0.5546	0.4442	403124	ebi-a-GCS	0.0068	0.0117	0.558799	0.4452	393873	ebi-a-GCST007091
rs1569723	A	C	0.1882	0.0344	4.56E-08	0.7293	2.69E+03	189998	Thyrotoxicosis with diffuse goitre	finn-b-E4_THYTOXG	-0.0039	0.0108	0.719	0.7544	403124	ebi-a-GCS	-0.015	0.0135	0.2673	0.7535	393873	ebi-a-GCST007091
rs179252	G	T	-0.3464	0.0311	8.67E-29	0.5902	1.17E+04	189998	Thyrotoxicosis with diffuse goitre	finn-b-E4_THYTOXG	-0.0214	0.0093	0.0215	0.4843	403124	ebi-a-GCS	0.0168	0.0117	0.1522	0.4848	393873	ebi-a-GCST007091
rs189424	G	A	-0.2232	0.032	3.07E-12	0.5129	4.85E+03	189998	Thyrotoxicosis with diffuse goitre	finn-b-E4_THYTOXG	-8.00E-04	0.0104	0.9376	0.4481	403124	ebi-a-GCS	-0.024	0.0134	0.07443	0.4479	393873	ebi-a-GCST007091
rs2395231	A	G	0.8393	0.0563	2.46E-50	0.09489	2.62E+04	189998	Thyrotoxicosis with diffuse goitre	finn-b-E4_THYTOXG	0.0417	0.0146	0.004308	0.1284	403124	ebi-a-GCS	0.0072	0.0188	0.7021	0.1282	393873	ebi-a-GCST007091
rs5872218	T	C	0.2147	0.0329	7.01E-11	0.3088	3.81E+03	189998	Thyrotoxicosis with diffuse goitre	finn-b-E4_THYTOXG	-0.0035	0.01	0.7229	0.312	403124	ebi-a-GCS	0.0171	0.0126	0.1739	0.3121	393873	ebi-a-GCST007091
rs6679677	A	C	0.4284	0.0448	1.22E-21	0.1434	8.97E+03	189998	Thyrotoxicosis with diffuse goitre	finn-b-E4_THYTOXG	0.0086	0.0153	0.575999	0.1016	403124	ebi-a-GCS	-0.0228	0.0193	0.2375	0.1012	393873	ebi-a-GCST007091
rs1103835	G	C	0.1932	0.029	2.77E-11	0.3066	3.08E+03	190799	Thyrotoxicosis, other and/or unspecified	finn-b-E4_THYTOXN	0.0029	0.0102	0.7739	0.2979	403124	ebi-a-GCS	-0.0157	0.0128	0.2194	0.2976	393873	ebi-a-GCST007091
rs121342	A	T	0.2154	0.038	1.46E-08	0.142	2.18E+03	190799	Thyrotoxicosis, other and/or unspecified	finn-b-E4_THYTOXN	0.0103	0.012	0.3903	0.1846	403124	ebi-a-GCS	0.0267	0.0151	0.076879	0.1842	393873	ebi-a-GCST007091
rs179453	G	T	0.5976	0.0475	2.99E-36	0.1013	1.33E+04	190799	Thyrotoxicosis, other and/or unspecified	finn-b-E4_THYTOXN	0.0412	0.0132	0.001729	0.146	403124	ebi-a-GCS	0.0181	0.0165	0.2748	0.1456	393873	ebi-a-GCST007091
rs433874	C	T	0.1905	0.0287	3.18E-11	0.3137	3.03E+03	190799	Thyrotoxicosis, other and/or unspecified	finn-b-E4_THYTOXN	-0.0055	0.0106	0.6058	0.2609	403124	ebi-a-GCS	-0.0137	0.0133	0.3017	0.2608	393873	ebi-a-GCST007091
rs4575545	A	G	0.1827	0.0289	2.54E-10	0.3051	2.74E+03	190799	Thyrotoxicosis, other and/or unspecified	finn-b-E4_THYTOXN	-0.005	0.0101	0.6216	0.3065	403124	ebi-a-GCS	0.0208	0.0127	0.1006	0.3067	393873	ebi-a-GCST007091
rs4903961	G	C	0.3003	0.0294	1.60E-24	0.2969	7.46E+03	190799	Thyrotoxicosis, other and/or unspecified	finn-b-E4_THYTOXN	0.0292	0.0096	0.002343	0.3757	403124	ebi-a-GCS	-0.0052	0.0121	0.6647	0.3751	393873	ebi-a-GCST007091
rs6679677	A	C	0.3393	0.0388	2.32E-18	0.1434	5.55E+03	190799	Thyrotoxicosis, other and/or unspecified	finn-b-E4_THYTOXN	0.0086	0.0153	0.575999	0.1016	403124	ebi-a-GCS	-0.0228	0.0193	0.2375	0.1012	393873	ebi-a-GCST007091
rs7289191	A	G	0.3717	0.0669	2.73E-08	0.04033	2.06E+03	190799	Thyrotoxicosis, other and/or unspecified	finn-b-E4_THYTOXN	0.0384	0.0344	0.2639	0.0193	403124	ebi-a-GCS	0.0188	0.0431	0.6623	0.0193	393873	ebi-a-GCST007091

SNP	effect_allele	other_allele	Exposure								Outcome						
			beta	se	pva	eaf	F-statistics	samplesize	name	id	beta	se	pva	eaf	samplesize	name	id
rs143384	G	A	-0.0935	0.0095	4.77E-23	0.4034	1.70E+03	403124	Knee OA	ebi-a-GCST007090	0.0753	0.0467	0.1066	0.4407	18258	Autoimmune hyperth	finn-b-AUTOIMMUNE_HYPERTHYROIDISM
rs4775006	A	C	0.0578	0.0094	8.40E-10	0.4114	6.53E+02	403124	Knee OA	ebi-a-GCST007090	-0.0219	0.0466	0.6376	0.4364	18258	Autoimmune hyperth	finn-b-AUTOIMMUNE_HYPERTHYROIDISM
rs5611684	A	G	0.0612	0.0097	3.19E-10	0.3563	6.94E+02	403124	Knee OA	ebi-a-GCST007090	-0.0374	0.0488	0.4428	0.344	18258	Autoimmune hyperth	finn-b-AUTOIMMUNE_HYPERTHYROIDISM
rs8067763	A	G	-0.0566	0.0095	2.39E-09	0.5936	6.24E+02	403124	Knee OA	ebi-a-GCST007090	0.022	0.049	0.6526	0.6632	18258	Autoimmune hyperth	finn-b-AUTOIMMUNE_HYPERTHYROIDISM
rs1078301	T	A	0.0679	0.0106	1.27E-10	0.2685	7.31E+02	403124	Knee OA	ebi-a-GCST007092	-0.0227	0.3884	0.3884	0.2624	217835	Thyrotoxicosis	finn-b-THYROTOXICOSIS
rs143384	G	A	-0.0935	0.0095	4.77E-23	0.4034	1.70E+03	403124	Knee OA	ebi-a-GCST007093	0.0576	0.0135101	0.0135101	0.4408	217835	Thyrotoxicosis	finn-b-THYROTOXICOSIS
rs4775006	A	C	0.0578	0.0094	8.40E-10	0.4114	6.53E+02	403124	Knee OA	ebi-a-GCST007094	-0.0015	0.9486	0.9486	0.4357	217835	Thyrotoxicosis	finn-b-THYROTOXICOSIS
rs5611684	A	G	0.0612	0.0097	3.19E-10	0.3563	6.94E+02	403124	Knee OA	ebi-a-GCST007095	-0.0053	0.827	0.827	0.344	217835	Thyrotoxicosis	finn-b-THYROTOXICOSIS
rs8067763	A	G	-0.0566	0.0095	2.39E-09	0.5936	6.24E+02	403124	Knee OA	ebi-a-GCST007096	0.0126	0.6071	0.6071	0.6635	217835	Thyrotoxicosis	finn-b-THYROTOXICOSIS
rs1078301	T	A	0.0679	0.0106	1.27E-10	0.2685	7.31E+02	403124	Knee OA	ebi-a-GCST007090	-0.0382	0.0346	0.2696	0.2627	189998	Thyrotoxicosis with d	finn-b-E4_THYTOXGOITDIF
rs143384	G	A	-0.0935	0.0095	4.77E-23	0.4034	1.70E+03	403124	Knee OA	ebi-a-GCST007090	0.0347	0.0307	0.2575	0.4407	189998	Thyrotoxicosis with d	finn-b-E4_THYTOXGOITDIF
rs4775006	A	C	0.0578	0.0094	8.40E-10	0.4114	6.53E+02	403124	Knee OA	ebi-a-GCST007090	0.0129	0.0306	0.673899	0.4362	189998	Thyrotoxicosis with d	finn-b-E4_THYTOXGOITDIF
rs5611684	A	G	0.0612	0.0097	3.19E-10	0.3563	6.94E+02	403124	Knee OA	ebi-a-GCST007090	-0.0261	0.032	0.4153	0.3442	189998	Thyrotoxicosis with d	finn-b-E4_THYTOXGOITDIF
rs8067763	A	G	-0.0566	0.0095	2.39E-09	0.5936	6.24E+02	403124	Knee OA	ebi-a-GCST007090	0	0.0322	0.9992	0.6633	189998	Thyrotoxicosis with d	finn-b-E4_THYTOXGOITDIF
rs1078301	T	A	0.0679	0.0106	1.27E-10	0.2685	7.31E+02	403124	Knee OA	ebi-a-GCST007090	-0.0325	0.0303	0.2831	0.2627	190799	Thyrotoxicosis, other	finn-b-E4_THYTOXNAS
rs143384	G	A	-0.0935	0.0095	4.77E-23	0.4034	1.70E+03	403124	Knee OA	ebi-a-GCST007090	0.0613	0.0268	0.02223	0.4409	190799	Thyrotoxicosis, other	finn-b-E4_THYTOXNAS
rs4775006	A	C	0.0578	0.0094	8.40E-10	0.4114	6.53E+02	403124	Knee OA	ebi-a-GCST007090	-0.0128	0.0267	0.6324	0.4361	190799	Thyrotoxicosis, other	finn-b-E4_THYTOXNAS
rs5611684	A	G	0.0612	0.0097	3.19E-10	0.3563	6.94E+02	403124	Knee OA	ebi-a-GCST007090	-0.0027	0.028	0.9225	0.3443	190799	Thyrotoxicosis, other	finn-b-E4_THYTOXNAS
rs8067763	A	G	-0.0566	0.0095	2.39E-09	0.5936	6.24E+02	403124	Knee OA	ebi-a-GCST007090	0.0085	0.0282	0.762801	0.6633	190799	Thyrotoxicosis, other	finn-b-E4_THYTOXNAS
rs1049236	T	G	0.1518	0.0148	1.25E-24	0.19	2.81E+03	393873	Hip OA	ebi-a-GCST007091	-0.1134	0.0613	0.0641195	0.1716	18258	Autoimmune hyperth	finn-b-AUTOIMMUNE_HYPERTHYROIDISM
rs1089601	A	G	-0.0782	0.0132	2.74E-09	0.2695	9.51E+02	393873	Hip OA	ebi-a-GCST007091	0.0013	0.0624	0.9831	0.1663	18258	Autoimmune hyperth	finn-b-AUTOIMMUNE_HYPERTHYROIDISM
rs1105909	T	C	0.0759	0.0117	7.38E-11	0.4777	1.14E+03	393873	Hip OA	ebi-a-GCST007091	0.0261	0.047	0.578699	0.4071	18258	Autoimmune hyperth	finn-b-AUTOIMMUNE_HYPERTHYROIDISM
rs1157405	C	T	0.1263	0.0224	1.60E-08	0.0741	8.64E+02	393873	Hip OA	ebi-a-GCST007091	-0.128	0.1272	0.314	0.03268	18258	Autoimmune hyperth	finn-b-AUTOIMMUNE_HYPERTHYROIDISM
rs1158364	T	C	-0.0811	0.0131	5.57E-10	0.2764	1.04E+03	393873	Hip OA	ebi-a-GCST007091	-0.0395	0.0498	0.4276	0.3201	18258	Autoimmune hyperth	finn-b-AUTOIMMUNE_HYPERTHYROIDISM
rs1220922	A	C	0.1558	0.0191	3.88E-16	0.1032	1.78E+03	393873	Hip OA	ebi-a-GCST007091	-0.0487	0.0676	0.4706	0.1353	18258	Autoimmune hyperth	finn-b-AUTOIMMUNE_HYPERTHYROIDISM
rs1330060	G	A	0.0716	0.0119	1.65E-09	0.4508	1.00E+03	393873	Hip OA	ebi-a-GCST007091	-0.0211	0.0465	0.650801	0.5291	18258	Autoimmune hyperth	finn-b-AUTOIMMUNE_HYPERTHYROIDISM
rs1835323	T	C	-0.0673	0.0123	4.56E-08	0.3428	8.05E+02	393873	Hip OA	ebi-a-GCST007091	0.0457	0.0474	0.3342	0.401	18258	Autoimmune hyperth	finn-b-AUTOIMMUNE_HYPERTHYROIDISM
rs1913707	G	A	-0.0795	0.012	2.96E-11	0.3877	1.19E+03	393873	Hip OA	ebi-a-GCST007091	-0.027	0.0469	0.5643	0.3963	18258	Autoimmune hyperth	finn-b-AUTOIMMUNE_HYPERTHYROIDISM
rs2396502	C	A	0.0842	0.012	2.12E-12	0.6018	1.34E+03	393873	Hip OA	ebi-a-GCST007091	-0.0531	0.0471	0.2595	0.5894	18258	Autoimmune hyperth	finn-b-AUTOIMMUNE_HYPERTHYROIDISM
rs2785988	A	C	0.0828	0.0127	7.30E-11	0.2988	1.13E+03	393873	Hip OA	ebi-a-GCST007091	-0.0032	0.0504	0.9489	0.299	18258	Autoimmune hyperth	finn-b-AUTOIMMUNE_HYPERTHYROIDISM
rs2836618	A	G	0.0876	0.0132	3.20E-11	0.2613	1.17E+03	393873	Hip OA	ebi-a-GCST007091	-0.0233	0.0503	0.6436	0.303	18258	Autoimmune hyperth	finn-b-AUTOIMMUNE_HYPERTHYROIDISM
rs3774355	A	G	0.0907	0.0121	8.20E-14	0.3601	1.50E+03	393873	Hip OA	ebi-a-GCST007091	0.06	0.0476	0.207	0.3742	18258	Autoimmune hyperth	finn-b-AUTOIMMUNE_HYPERTHYROIDISM
rs4338381	G	A	-0.095	0.0121	4.37E-15	0.3681	1.66E+03	393873	Hip OA	ebi-a-GCST007091	-0.0293	0.0467	0.53	0.4282	18258	Autoimmune hyperth	finn-b-AUTOIMMUNE_HYPERTHYROIDISM
rs6206328	G	A	0.0964	0.014	5.30E-12	0.2229	1.27E+03	393873	Hip OA	ebi-a-GCST007091	0.0318	0.0841	0.705601	0.07966	18258	Autoimmune hyperth	finn-b-AUTOIMMUNE_HYPERTHYROIDISM
rs7222178	A	T	0.0965	0.0146	3.77E-11	0.1991	1.17E+03	393873	Hip OA	ebi-a-GCST007091	0.0883	0.0666	0.1845	0.1395	18258	Autoimmune hyperth	finn-b-AUTOIMMUNE_HYPERTHYROIDISM
rs7476779	G	A	-0.0751	0.0126	2.56E-09	0.3171	9.64E+02	393873	Hip OA	ebi-a-GCST007091	0.0278	0.0479	0.5623	0.3682	18258	Autoimmune hyperth	finn-b-AUTOIMMUNE_HYPERTHYROIDISM
rs7571789	C	T	-0.0886	0.0117	3.26E-14	0.5239	1.55E+03	393873	Hip OA	ebi-a-GCST007091	0.0163	0.0468	0.728299	0.574	18258	Autoimmune hyperth	finn-b-AUTOIMMUNE_HYPERTHYROIDISM
rs7905604	G	A	0.1625	0.0268	1.33E-09	0.0503	9.96E+02	393873	Hip OA	ebi-a-GCST007091	-0.0017	0.0997	0.9862	0.05729	18258	Autoimmune hyperth	finn-b-AUTOIMMUNE_HYPERTHYROIDISM
rs798748	C	T	0.0715	0.012	2.50E-09	0.6183	9.53E+02	393873	Hip OA	ebi-a-GCST007091	0.0012	0.0503	0.9802	0.7028	18258	Autoimmune hyperth	finn-b-AUTOIMMUNE_HYPERTHYROIDISM
rs8028769	G	A	0.1093	0.0184	2.66E-09	0.1131	9.46E+02	393873	Hip OA	ebi-a-GCST007091	-0.0351	0.0821	0.669501	0.09031	18258	Autoimmune hyperth	finn-b-AUTOIMMUNE_HYPERTHYROIDISM
rs1049236	T	G	0.1518	0.0148	1.25E-24	0.19	2.81E+03	393873	Hip OA	ebi-a-GCST007091	-0.0146	0.0307	0.6343	0.171	217835	Thyrotoxicosis	finn-b-THYROTOXICOSIS
rs1089601	A	G	-0.0782	0.0132	2.74E-09	0.2695	9.51E+02	393873	Hip OA	ebi-a-GCST007091	-0.0385	0.0311	0.2161	0.1666	217835	Thyrotoxicosis	finn-b-THYROTOXICOSIS
rs1105909	T	C	0.0759	0.0117	7.38E-11	0.4777	1.14E+03	393873	Hip OA	ebi-a-GCST007091	7.00E-04	0.0235	0.9755	0.4075	217835	Thyrotoxicosis	finn-b-THYROTOXICOSIS
rs1157405	C	T	0.1263	0.0224	1.60E-08	0.0741	8.64E+02	393873	Hip OA	ebi-a-GCST007091	0.1101	0.064	0.0853395	0.03265	217835	Thyrotoxicosis	finn-b-THYROTOXICOSIS
rs1158364	T	C	-0.0811	0.0131	5.57E-10	0.2764	1.04E+03	393873	Hip OA	ebi-a-GCST007091	0.0079	0.0249	0.7514	0.3199	217835	Thyrotoxicosis	finn-b-THYROTOXICOSIS
rs1204094	T	C	-0.0665	0.012	2.83E-08	0.3843	8.26E+02	393873	Hip OA	ebi-a-GCST007091	0.0398	0.0235	0.089809	0.4145	217835	Thyrotoxicosis	finn-b-THYROTOXICOSIS
rs1220922	A	C	0.1558	0.0191	3.88E-16	0.1032	1.78E+03	393873	Hip OA	ebi-a-GCST007091	-0.0452	0.0338	0.1814	0.135	217835	Thyrotoxicosis	finn-b-THYROTOXICOSIS
rs1330060	G	A	0.0716	0.0119	1.65E-09	0.4508	1.00E+03	393873	Hip OA	ebi-a-GCST007091	0.0191	0.0233	0.4122	0.5286	217835	Thyrotoxicosis	finn-b-THYROTOXICOSIS
rs1835323	T	C	-0.0673	0.0123	4.56E-08	0.3428	8.05E+02	393873	Hip OA	ebi-a-GCST007091	0.0459	0.0236	0.0522204	0.4013	217835	Thyrotoxicosis	finn-b-THYROTOXICOSIS
rs1913707	G	A	-0.0795	0.012	2.96E-11	0.3877	1.19E+03	393873	Hip OA	ebi-a-GCST007091	-0.0209	0.0235	0.3745	0.3967	217835	Thyrotoxicosis	finn-b-THYROTOXICOSIS
rs2396502	C	A	0.0842	0.012	2.12E-12	0.6018	1.34E+03	393873	Hip OA	ebi-a-GCST007091	-0.0215	0.0235	0.3616	0.589	217835	Thyrotoxicosis	finn-b-THYROTOXICOSIS
rs2785988	A	C	0.0828	0.0127	7.30E-11	0.2988	1.13E+03	393873	Hip OA	ebi-a-GCST007091	0.0074	0.0252	0.7703	0.2988	217835	Thyrotoxicosis	finn-b-THYROTOXICOSIS
rs2836618	A	G	0.0876	0.0132	3.20E-11	0.2613	1.17E+03	393873	Hip OA	ebi-a-GCST007091	-0.0464	0.0251	0.0648097	0.3033	217835	Thyrotoxicosis	finn-b-THYROTOXICOSIS
rs3774355	A	G	0.0907	0.0121	8.20E-14	0.3601	1.50E+03	393873	Hip OA	ebi-a-GCST007091	0.0165	0.0238	0.4884	0.3736	217835	Thyrotoxicosis	finn-b-THYROTOXICOSIS
rs4252548	T	C	0.2785	0.0396	1.96E-12	0.022	1.32E+03	393873	Hip OA	ebi-a-GCST007091	0.1536	0.0725	0.0340596	0.02558	217835	Thyrotoxicosis	finn-b-THYROTOXICOSIS
rs4338381	G	A	-0.095	0.0121	4.37E-15	0.3681	1.66E+03	393873	Hip OA	ebi-a-GCST007091	-0.0012	0.0234	0.9587	0.4281	217835	Thyrotoxicosis	finn-b-THYROTOXICOSIS
rs6206328	G	A	0.0964	0.014	5.30E-12	0.2229	1.27E+03	393873	Hip OA	ebi-a-GCST007091	-0.0172	0.0422	0.682501	0.08001	217835	Thyrotoxicosis	finn-b-THYROTOXICOSIS
rs7222178	A	T	0.0965	0.0146	3.77E-11	0.1991	1.17E+03	393873	Hip OA	ebi-a-GCST007091	-0.0249	0.0334	0.4564	0.1392	217835	Thyrotoxicosis	finn-b-THYROTOXICOSIS
rs7476779	G	A	-0.0751	0.0126	2.56E-09	0.3171	9.64E+02	393873	Hip OA	ebi-a-GCST007091	0.0042	0.024	0.8611	0.3677	217835	Thyrotoxicosis	finn-b-THYROTOXICOSIS
rs7571789	C	T	-0.0886	0.0117	3.26E-14	0.5239	1.55E+03	393873	Hip OA	ebi-a-GCST007091	0.0296	0.0234	0.2055	0.5747	217835	Thyrotoxicosis	finn-b-THYROTOXICOSIS
rs7905604	G	A	0.1625	0.0268	1.33E-09	0.0503	9.96E+02	393873	Hip OA	ebi-a-GCST007091	-0.0141	0.05	0.777499	0.05736	217835	Thyrotoxicosis	finn-b-THYROTOXICOSIS
rs798748	C	T	0.0715	0.012	2.50E-09	0.6183	9.53E+02	393873	Hip OA	ebi-a-GCST007091	0.0202	0.0252	0.4226	0.7035	217835	Thyrotoxicosis	finn-b-THYROTOXICOSIS
rs8028769	G	A	0.1093	0.0184	2.66E-09	0.1131	9.46E+02	393873	Hip OA	ebi-a-GCST007091	0.0155	0.041	0.706401	0.09007	217835	Thyrotoxicosis	finn-b-THYROTOXICOSIS
rs1049236	T	G	0.1518	0.0148	1.25E-24	0.19	2.81E+03	393873	Hip OA	ebi-a-GCST007091	-0.0491	0.0403	0.2231	0.1713	189998	Thyrotoxicosis with d	finn-b-E4_THYTOXGOITDIF
rs1089601	A	G	-0.0782	0.0132	2.74E-09	0.2695	9.51E+02	393873	Hip OA	ebi-a-GCST007091	-0.0135	0.0409	0.740901	0.1663	189998	Thyrotoxicosis with d	finn-b-E4_THYTOXGOITDIF
rs1105909	T	C	0.0759	0.0117	7.38E-11	0.4777	1.14E+03	393873	Hip OA	ebi-a-GCST007091	0.0075	0.0308	0.8072	0.4072	189998	Thyrotoxicosis with d	finn-b-E4_THYTOXGOITDIF
rs1157405	C	T	0.1263	0.0224	1.60E-08	0.0741	8.64E+02	393873	Hip OA	ebi-a-GCST007091	0.0458	0.0841	0.586001	0.03252	189998	Thyrotoxicosis with d	finn-b-E4_THYTOXGOITDIF
rs1158364	T	C	-0.0811	0.0131	5.57E-10	0.2764	1.04E+03	393873	Hip OA	ebi-a-GCST007091	-0.0167	0.0327	0.609399	0.3199	189998	Thyrotoxicosis with d	finn-b-E4_THYTOXGOITDIF
rs1204094	T	C	-0.0665	0.012	2.83E-08	0.3843	8.26E+02	393873	Hip OA	ebi-a-GCST007091	0.0448	0.0309	0.147	0.4141	189998	Thyrotoxicosis with d	finn-b-E4_THYTOXGOITDIF
rs1220922	A	C	0.1558	0.0191	3.88E-16	0.1032	1.78E+03	393873	Hip OA	ebi-a-GCST007091	-0.0856	0.0445	0.0544703	0.1351	189998	Thyrotoxicosis with d	finn-b-E4_THYTOXGOITDIF
rs1330060	G	A	0.0716	0.0119	1.65E-09	0.4508	1.00E+03	393873	Hip OA	ebi-a-GCST007091	0.0067	0.0306	0.8253	0.5287	189998	Thyrotoxicosis with d	finn-b-E4_THYTOXGOITDIF
rs1835323	T	C	-0.0673	0.0123	4.56E-08	0.3428	8.05E+02	393873	Hip OA	ebi-a-GCST007091	0.0465	0.031	0.1337	0.4007	189998	Thyrotoxicosis with d	finn-b-E4_THYTOXGOITDIF
rs1913707	G	A	-0.0795	0.012	2.96E-11	0.3877	1.19E+03	393873	Hip OA	ebi-a-GCST007091	-0.0262	0.0308	0.3957	0.3965	189998	Thyrotoxicosis with d	finn-b-E4_THYTOXGOITDIF
rs2396502	C	A	0.0842	0.012	2.12E-12	0.6018	1.34E+03	393873	Hip OA	ebi-a-GCST007091	-0.0372	0.0309	0.2289	0.5892	189998	Thyrotoxicosis with d	finn-b-E4_THYTOXGOITDIF
rs2785988	A	C	0.0828	0.0127	7.30E-11	0.2988	1.13E+03	393873	Hip OA	ebi-a-GCST007091	0.014	0.0332	0.6738	0.2991	189998	Thyrotoxicosis with d	finn-b-E4_THYTOXGOITDIF
rs2836618	A	G	0.0876	0.0132	3.20E-11	0.2613	1.17E+03	393873	Hip OA	ebi-a-GCST007091	-0.048	0.033	0.1463	0.3032	189998	Thyrotoxicosis with d	finn-b-E4_THYTOXGOITDIF
rs3774355	A	G	0.0907	0.0121	8.20E-14	0.3601	1.50E+03	393873	Hip OA	ebi-a-GCST007091	0.0265	0.0313	0.3964	0.3742	189998	Thyrotoxicosis with d	finn-b-E4_THYTOXGOITDIF
rs4252548	T	C	0.2785	0.0396	1.96E-12	0.022	1.32E+03	393873	Hip OA	ebi-a-GCST007091	0.2096	0.095	0.0273697	0.02557	189998	Thyrotoxicosis with d	finn-b-E4_THYTOXGOITDIF
rs4338381	G	A	-0.095	0.0121	4.37E-15	0.3681	1.66E+03	393873	Hip OA	ebi-a-GCST007091	0.0018	0.0307	0.9528	0.4282	189998	Thyrotoxicosis with d	finn-b-E4_THYTOXGOITDIF
rs6206328	G	A	0.0964	0.014	5.30E-12	0.2229	1.27E+03	393873	Hip OA	ebi-a-GCST007091	5.00E-04	0.0555	0.9933	0.07958	189998	Thyrotoxicosis with d	finn-b-E4_THYTOXGOITDIF
rs7222178	A	T	0.0965	0.0146	3.77E-11	0.1991	1.17E+03	393873	Hip OA	ebi-a-GCST007091	-0.0426	0.0439	0.3311	0.1394	189998	Thyrotoxicosis with d	finn-b-E4_THYTOXGOITDIF
rs7476779	G	A	-0.0751	0.0126	2.56E-09	0.3171	9.64E+02	393873	Hip OA	ebi-a-GCST007091	-0.0017	0.0315	0.956	0.3683	189998	Thyrotoxicosis with d	finn-b-E4_THYTOXGOITDIF

rs7571789	C	T	-0.0886	0.0117	3.26E-14	0.5239	1.55E+03	393873	Hip OA	ebi-a-GCST007091	0.0238	0.0307	0.4382	0.5745	189998	Thyrotoxicosis with d	finn-b-E4_THYTOXGOITDIF
rs7905604	G	A	0.1625	0.0268	1.33E-09	0.0503	9.96E+02	393873	Hip OA	ebi-a-GCST007091	0.0109	0.0653	0.8673	0.0575	189998	Thyrotoxicosis with d	finn-b-E4_THYTOXGOITDIF
rs798748	C	T	0.0715	0.012	2.50E-09	0.6183	9.53E+02	393873	Hip OA	ebi-a-GCST007091	-0.0107	0.0331	0.7462	0.703	189998	Thyrotoxicosis with d	finn-b-E4_THYTOXGOITDIF
rs8028769	G	A	0.1093	0.0184	2.66E-09	0.1131	9.46E+02	393873	Hip OA	ebi-a-GCST007091	0.0474	0.0539	0.379	0.09015	189998	Thyrotoxicosis with d	finn-b-E4_THYTOXGOITDIF
rs1049236	T	G	0.1518	0.0148	1.25E-24	0.19	2.81E+03	393873	Hip OA	ebi-a-GCST007091	-0.0147	0.0352	0.6751	0.1713	190799	Thyrotoxicosis, other	finn-b-E4_THYTOXNAS
rs1089601	A	G	-0.0782	0.0132	2.74E-09	0.2695	9.51E+02	393873	Hip OA	ebi-a-GCST007091	-0.0588	0.0358	0.0999494	0.1663	190799	Thyrotoxicosis, other	finn-b-E4_THYTOXNAS
rs1105909	T	C	0.0759	0.0117	7.38E-11	0.4777	1.14E+03	393873	Hip OA	ebi-a-GCST007091	0.0342	0.027	0.2049	0.4073	190799	Thyrotoxicosis, other	finn-b-E4_THYTOXNAS
rs1157405	C	T	0.1263	0.0224	1.60E-08	0.0741	8.64E+02	393873	Hip OA	ebi-a-GCST007091	0.098	0.0735	0.1825	0.03256	190799	Thyrotoxicosis, other	finn-b-E4_THYTOXNAS
rs1158364	T	C	-0.0811	0.0131	5.57E-10	0.2764	1.04E+03	393873	Hip OA	ebi-a-GCST007091	0.0194	0.0286	0.4972	0.32	190799	Thyrotoxicosis, other	finn-b-E4_THYTOXNAS
rs1204094	T	C	-0.0665	0.012	2.83E-08	0.3843	8.26E+02	393873	Hip OA	ebi-a-GCST007091	0.0406	0.027	0.1332	0.4141	190799	Thyrotoxicosis, other	finn-b-E4_THYTOXNAS
rs1220922	A	C	0.1558	0.0191	3.88E-16	0.1032	1.78E+03	393873	Hip OA	ebi-a-GCST007091	-0.0485	0.0389	0.2121	0.1351	190799	Thyrotoxicosis, other	finn-b-E4_THYTOXNAS
rs1330060	G	A	0.0716	0.0119	1.65E-09	0.4508	1.00E+03	393873	Hip OA	ebi-a-GCST007091	0.0215	0.0267	0.4206	0.5287	190799	Thyrotoxicosis, other	finn-b-E4_THYTOXNAS
rs1835323	T	C	-0.0673	0.0123	4.56E-08	0.3428	8.05E+02	393873	Hip OA	ebi-a-GCST007091	0.0402	0.0272	0.1387	0.4007	190799	Thyrotoxicosis, other	finn-b-E4_THYTOXNAS
rs1913707	G	A	-0.0795	0.012	2.96E-11	0.3877	1.19E+03	393873	Hip OA	ebi-a-GCST007091	-0.04	0.027	0.1378	0.3964	190799	Thyrotoxicosis, other	finn-b-E4_THYTOXNAS
rs2396502	C	A	0.0842	0.012	2.12E-12	0.6018	1.34E+03	393873	Hip OA	ebi-a-GCST007091	-0.0284	0.027	0.2931	0.5892	190799	Thyrotoxicosis, other	finn-b-E4_THYTOXNAS
rs2785988	A	C	0.0828	0.0127	7.30E-11	0.2988	1.13E+03	393873	Hip OA	ebi-a-GCST007091	-0.0114	0.029	0.692901	0.2991	190799	Thyrotoxicosis, other	finn-b-E4_THYTOXNAS
rs2836618	A	G	0.0876	0.0132	3.20E-11	0.2613	1.17E+03	393873	Hip OA	ebi-a-GCST007091	-0.0437	0.0289	0.1298	0.3032	190799	Thyrotoxicosis, other	finn-b-E4_THYTOXNAS
rs3774355	A	G	0.0907	0.0121	8.20E-14	0.3601	1.50E+03	393873	Hip OA	ebi-a-GCST007091	0.021	0.0273	0.4433	0.3743	190799	Thyrotoxicosis, other	finn-b-E4_THYTOXNAS
rs4252548	T	C	0.2785	0.0396	1.96E-12	0.022	1.32E+03	393873	Hip OA	ebi-a-GCST007091	0.2216	0.083	0.00757304	0.0256	190799	Thyrotoxicosis, other	finn-b-E4_THYTOXNAS
rs4338381	G	A	-0.095	0.0121	4.37E-15	0.3681	1.66E+03	393873	Hip OA	ebi-a-GCST007091	-0.0175	0.0268	0.514801	0.4281	190799	Thyrotoxicosis, other	finn-b-E4_THYTOXNAS
rs6206328	G	A	0.0964	0.014	5.30E-12	0.2229	1.27E+03	393873	Hip OA	ebi-a-GCST007091	-0.0141	0.0485	0.770801	0.07958	190799	Thyrotoxicosis, other	finn-b-E4_THYTOXNAS
rs7222178	A	T	0.0965	0.0146	3.77E-11	0.1991	1.17E+03	393873	Hip OA	ebi-a-GCST007091	0.0046	0.0382	0.9041	0.1395	190799	Thyrotoxicosis, other	finn-b-E4_THYTOXNAS
rs7476779	G	A	-0.0751	0.0126	2.56E-09	0.3171	9.64E+02	393873	Hip OA	ebi-a-GCST007091	0.0045	0.0275	0.8696	0.3683	190799	Thyrotoxicosis, other	finn-b-E4_THYTOXNAS
rs7571789	C	T	-0.0886	0.0117	3.26E-14	0.5239	1.55E+03	393873	Hip OA	ebi-a-GCST007091	0.0244	0.0269	0.3637	0.5745	190799	Thyrotoxicosis, other	finn-b-E4_THYTOXNAS
rs7905604	G	A	0.1625	0.0268	1.33E-09	0.0503	9.96E+02	393873	Hip OA	ebi-a-GCST007091	0.019	0.0571	0.7387	0.05751	190799	Thyrotoxicosis, other	finn-b-E4_THYTOXNAS
rs798748	C	T	0.0715	0.012	2.50E-09	0.6183	9.53E+02	393873	Hip OA	ebi-a-GCST007091	0.0328	0.029	0.2572	0.7031	190799	Thyrotoxicosis, other	finn-b-E4_THYTOXNAS
rs8028769	G	A	0.1093	0.0184	2.66E-09	0.1131	9.46E+02	393873	Hip OA	ebi-a-GCST007091	-0.0039	0.0473	0.9345	0.09009	190799	Thyrotoxicosis, other	finn-b-E4_THYTOXNAS
